# Facial Asymmetry in the Helical Grooves of Chiral Helical Polymers to Create 2D Single-Chain Archimedean Spiral Nanostructures

**DOI:** 10.1021/acsnano.5c10476

**Published:** 2025-09-10

**Authors:** Juan José Tarrío, Francisco Rey-Tarrío, Borja Hermida, Berta Fernández, Jeanne Crassous, Emilio Quiñoá, Rafael Rodríguez, Félix Freire

**Affiliations:** a CINBIO and Departamento de Química Orgánica. Campus Lagoas-Marcosende, 16784 Universidade de Vigo, Vigo E-36310, Spain; b Centro Singular de investigación en Química Biolóxica e Materiais Moleculares (CiQUS) and Departamento de Química Orgánica, 16780 Universidade de Santiago de Compostela, Santiago de Compostela E-15782, Spain; c Departamento de Química Física, 16780 Universidade de Santiago de Compostela, Santiago de Compostela E-15782, Spain; d Univ Rennes, CNRS, ISCR (Institut des Sciences Chimiques de Rennes) − UMR 6226, Rennes F-35000, France

**Keywords:** self-assembly, helical polymer, Archimedean nanospirals, aromatic substitution pattern, atomic force microscopy

## Abstract

Archimedean spirals are architectural motifs that are found in nature. The facial asymmetry of amphiphilic molecules or macromolecules has been a key parameter in the preparation of these well-organized two-dimensional nanostructures in the laboratory. This facial asymmetry is also present in the helical grooves of chiral helical *meta-*substituted poly­(phenylacetylene)­s (PPAs) and poly­(diphenylacetylene)­s (PDPAs), making them excellent candidates for self-assembly into 2D Archimedean nanospirals or nanotoroids. The facial asymmetry of the helix groove, with different polarities and hydrophobic/hydrophilic behaviors, impacts the self-assembly of *meta*-PPAs and *meta*-PDPAs compared to their *para*-substituted counterparts, which possess facial symmetry in the helix grooves. As a result, while *para*-substituted PPAs and *para*-substituted PDPAs self-assemble by drop-casting on highly oriented pyrolytic graphite to form 2D crystals via parallel packing of helical polymer chains, *meta*-substituted helical polymers undergo intramolecular self-assembly to create a 2D chiral Archimedean spiral nanostructure from a single polymer chain. The structural parameters obtained for the helical polymer in the 2D crystal and 2D chiral Archimedean spiral nanostructures are identical, indicating that the secondary structure of the polymer remains unchanged in both 2D nanomaterials. This finding regarding the self-assembly of the helical polymer into 2D chiral Archimedean spiral nanostructures allows the preparation of chiral nanostructures with potential applications in asymmetric catalysis, molecular recognition, and other cutting-edge applications.

## Graphical abstract

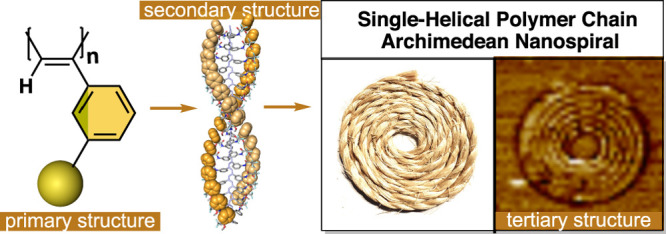

Archimedean nanospirals —spiral-shaped nanostructures characterized by constant radius and interlayer spacing— represent a highly rare and architecturally distinctive motif. These spirals are found in nature in living organisms such as ferns, millipedes, cabbages, human fingerprints, and so on. However, preparing them in a laboratory through molecular or macromolecular self-assembly is not an easy task.^1–3^ From the literature, it is known that these assemblies are typically formed from amphiphilic molecules or macromolecules exhibiting facial asymmetry. Notable examples include block copolymers,^4,5^ amphiphilic peptides,^6^ droplets,^7^ and supramolecular polymers based on porphyrins^8^ or azaacenes,^9^ among others.^10,11^ In all cases, the molecule or macromolecule has two different hydrophobic/hydrophilic terminal ends, which direct their supramolecular aggregation into high-order supramolecular structures, including 2D chiral Archimedean spirals (micro or nano). In this work, we want to go a step further and demonstrate that this type of supramolecular arrangement can also be obtained by the intramolecular self-assembly of single polymer chains. More precisely, we will show that an Archimedean spiral can be attained by intrachain interactions of a chiral helical polymer, such as poly­(acetylene)­s (PPAs) or poly­(diphenylacetylene)­s (PDPAs), whose helical sense or elongation can be tamed by the action of external stimuli.^12–16^ In these families of helical polymers, the periphery of the helix, and therefore the helical grooves, can be modulated by the aromatic substitution pattern: *para*, *meta*, and *ortho*.^17–19^ Interestingly, while *para*-substituted PPAs or PDPAs are widely studied in the literature, their *meta* and *ortho* counterparts are scarcely studied.^17–20^ Recently, it was found that chiral *meta*-monosubstituted PPAs or PDPAs generate well-folded *P* or *M* helical structures depending on the chirality of the monomer repeating unit. This screw sense induction is possible due to the formation of a stereoregular helix with a preferred conformation at the different dihedral angles of the macromolecule.^17–21^ Interestingly, the helices of *meta*-substituted PPAs and PDPAs exhibit a structural feature not observed in their *para*-substituted counterparts. Thus, while in *para*-PPAs and PDPAs the chiral pendant group is located at the periphery of the helix, producing a symmetric environment around the pendant (Scheme 1), in their *meta-*substituted counterparts, the asymmetry introduced in the aryl ring is transferred to their macromolecular helical scaffolds.

**Scheme 1:**
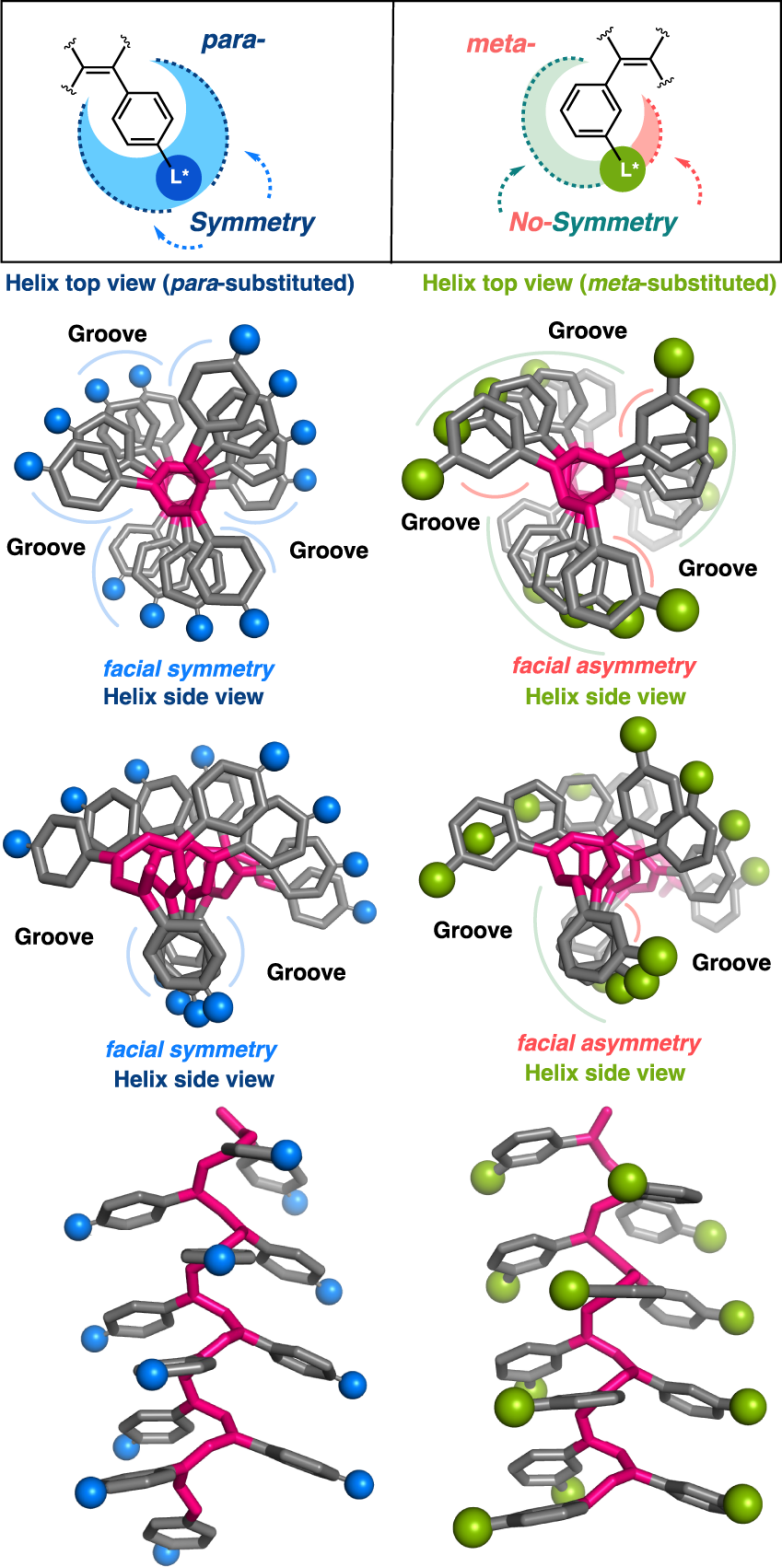
Structural Effects in *Para-* and *Meta-*substituted Aromatic Poly­(phenylacetylene)­s: Views from Different Perspectives Are Shown

This fact produces helical grooves with hydrophobic/hydrophilic facial asymmetry (Scheme 1), which could favor their self-assembly properties into 2D chiral Archimedean spiral nanostructures. Therefore, in this work, by using chiral *para*- and *meta*-substituted PPAs and chiral *para*- and *meta*-substituted PDPAs, we will demonstrate how the aromatic substitution pattern affects the morphology of the 2D aggregates generated by the self-assembly of these polymers once drop-cast on highly oriented pyrolytic graphite (HOPG). Thus, while in chiral *para*-substituted PPAs and PDPAs the parallel packing of the helical polymer chains is favored to form 2D crystals,^21–34^ in *meta*-substituted ones, 2D chiral Archimedean spiral nanostructures are obtained from intramolecular self-assembly of a single polymer chain. Both aggregates, 2D crystals and 2D chiral Archimedean spiral nanostructures, produce high-resolution AFM images that allow the extraction of structural parameters such as the pitch, sense, or width of the helix.^31–33^

## Results and Discussion

The *para*- and *meta*-substituted PPAs bearing the anilide of (*R*)-methoxy­(trifluoromethyl)­phenylacetic acid as a pendant [*p*-poly­(*R*)-**1** and *m-*poly­(*R*)-**1**] ^34–38^ and the *para*- and *meta*-substituted PDPAs bearing the benzamide of (*S*)-alanine methyl ester as pendant [*p*-poly­(*S*)-**2** and *m-*poly­(*S*)-**2**] were chosen as model compounds to carry out these studies (Figure 1).^39–42^ These polymers were prepared following well-established synthetic protocols described in the literature (see SI for synthetic details).

**Figure 1:**
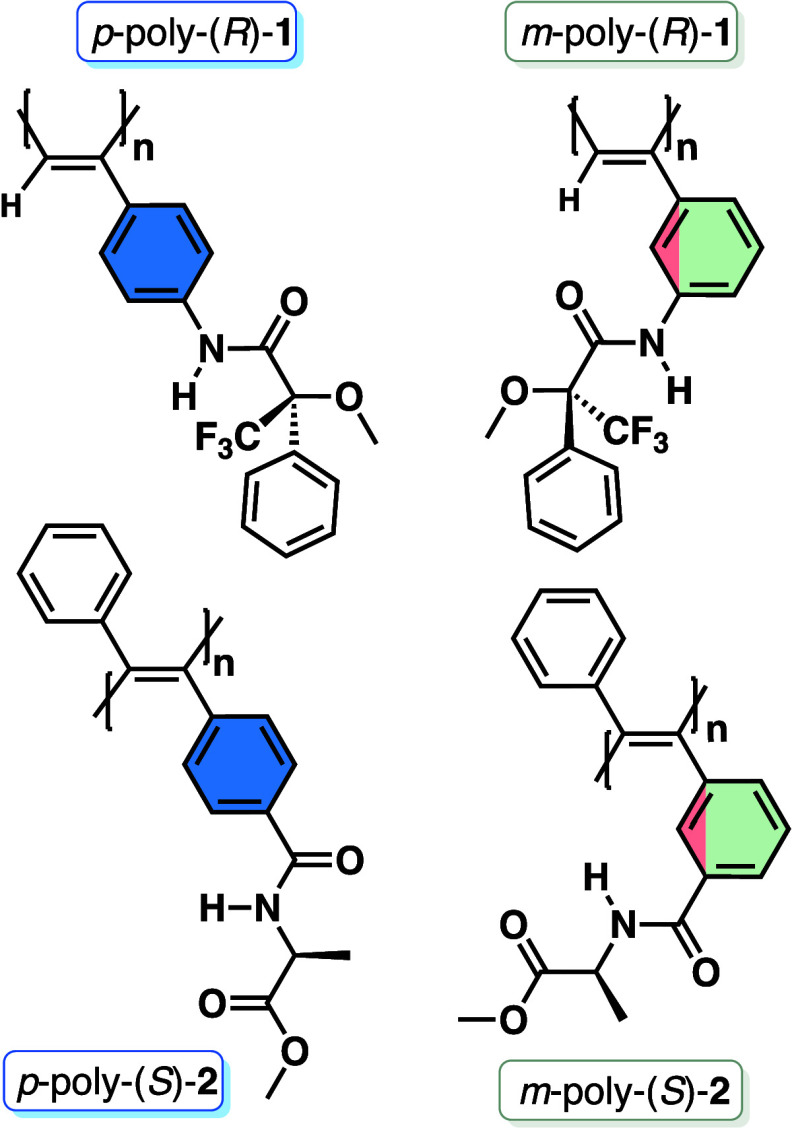
Chemical structures of *p*- and *m*-poly­(*R*)-**1** (PPA) and *p*- and *m*-poly­(*S*)-**2** (PDPA).

ECD studies of *p*-poly­(*R*)-**1** and *m-*poly­(*R*)-**1** (PPAs) in different solvents (*c* = 0.9 mM) show different dynamic behaviors depending on the aromatic substitution pattern (Figure 2 and Figure S5). Thus, while the *P*/*M* helical sense and screw sense excess of *p*-poly­(*R*)-**1** can be altered in solvents with different polarity or donor/acceptor ability [ECD band at 390 nm in THF (low polar/donor) ECD_390 (THF)_ > 0, *P*_helix_; ECD band at 390 nm in CHCl_3_ (low polar/nondonor) ECD_390 (CHCl3)_ < 0, *M*_helix_,; Figure 2a and Figure S5], the *P*/*M* helical sense of *m*-poly­(*R*)-**1** cannot be switched (Figure 2b and Figure S5). In the latter, a *P* screw sense is present in all solvents (Figure 2b), although in this case, an equilibrium is observed between two scaffolds with different elongations. For instance, in DMSO, *m*-poly­(*R*)-**1** shows an ECD_340(DMSO)_ > 0 and an ECD_420(DMSO)_ > 0, which correspond to the presence of a *P*_helix compressed_ and *P*_helix stretched_, respectively.^17–19^ An analogous stimuli-responsiveness associated with the aromatic substitution pattern is observed for *p*-poly­(*S*)-**2** and *m*-poly­(*S*)-**2** PDPAs. Thus, while *p*-poly­(*S*)-**2** shows a dynamic *P*/*M* helical behavior [ECD band at 394 nm in DMSO (high polar/donor) ECD_394 (DMSO)_ > 0, *M*_helix_; ECD band at 394 nm in CHCl_3_ (low polar/nondonor) ECD_394 (CHCl3)_ < 0, *P*_helix_; Figure 2c and Figure S5], *m-*poly­(*S*)-**2** shows a quasi-static one after thermal annealing at 80 °C for 24h—an approach that is commonly employed in this family of polymers— ^39–53^ where almost identical ECD spectra at 387 nm are obtained in all solvents, i.e., ECD_387_ > 0, *M*_helix_ (Figure 2d and Figure S5).

**Figure 2:**
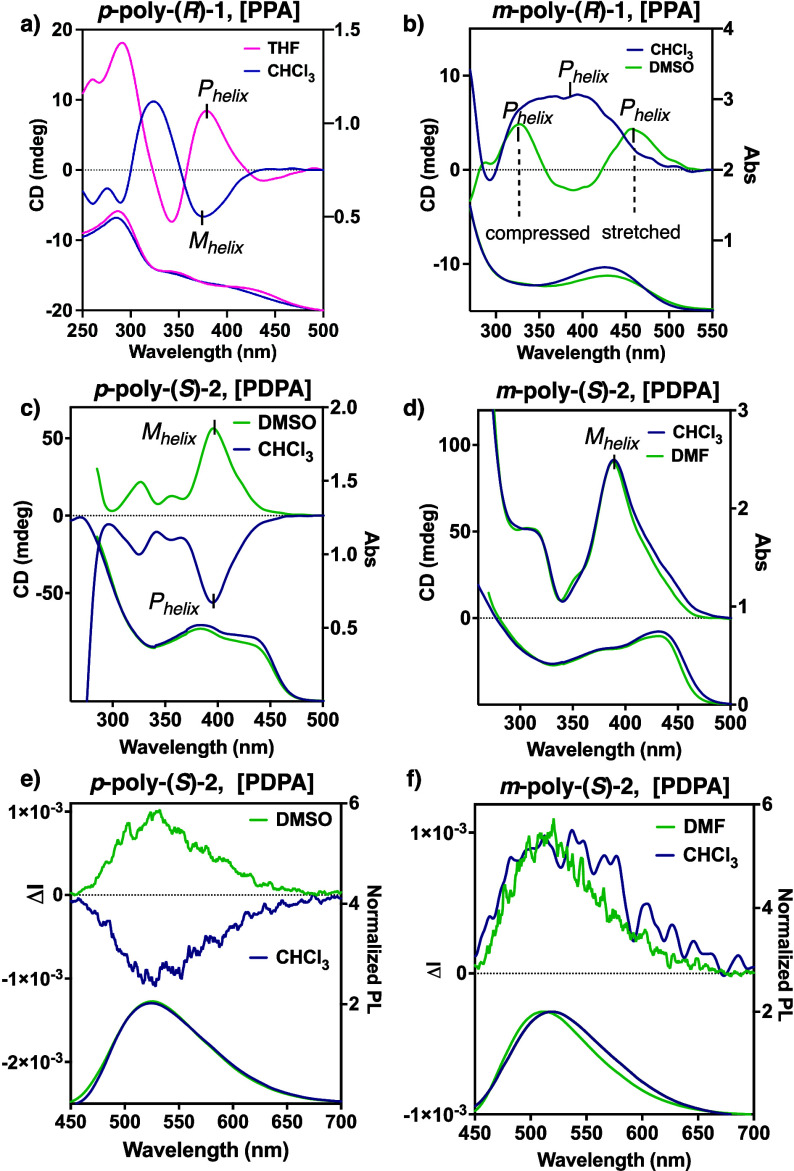
ECD and UV–vis spectra of (a) *p*-poly­(*R*)-**1** and (b) *m*-poly­(*R*)-**1** (PPAs, *c* = 0.9 mM) and (c) *p*-poly­(*S*)-**2** and (d) *m*-poly­(*S*)-**2** (PDPAs) (*c* = 1.6 mM). CPL and PL spectra of (e) *p*-poly­(*S*)-**2** and (f) *m*-poly­(*S*)-**2** (λ_Exc_ = 365 nm, *c* = 1.6 mM).

These ECD studies show therefore that *m*-poly­(*R*)-**1** (PPA) and *m*-poly­(*S*)-**2** (PDPA) cannot act as screw sense switches as occurs in their *para*-substituted counterparts. This difference in dynamic helical behavior between *para-* and *meta*-substituted PPAs and PDPAs is attributed to the steric hindrance arising from placing the pendant group closer to the polyene backbone (*para*-to-*meta* mutation). Interestingly, the maximum *g*_abs_ [*g*_abs_ = ECD/(UV–vis·32,984)] for *p*-poly­(*R*)-**1** is −3.7·10^–3^ (λ = 382 nm), and for *m-*poly­(*R*)-**1** (PPAs) in DMSO, it is +3.8·10^–4^ (λ = 325 nm, compressed helix) and +3.4·10^–4^ (λ = 470 nm, stretched helix), whereas those for *p*-poly­(*S*)-**2** and *m-*poly­(*S*)-**2** (PDPAs) are 5.3·10^–3^ and 6.3·10^–3^ at λ = 394 and λ = 387 nm, respectively. Photoluminescence (PL) studies were then carried out for the PDPAs [*p-*poly­(*S*)-**2** and *m-*poly­(*S*)-**2**] due to the light emission properties of this family of helical polymers [*p-*poly­(*S*)-**2**: ϕ_PL_ = 0.57 in DMSO, 0.26 in CHCl_3_; *m-*poly­(*S*)-**2**: ϕ_PL_ = 0.58 in DMSO, 0.25 in CHCl_3_], indicating similar emission properties for both PDPAs. Moreover, circularly polarized light (CPL) emission spectra were recorded for both *para*- and *meta*-poly­(*S*)-**2**, showing a maximum *g*_lum_ value of ca. ±1 × 10^–3^ obtained at 520 nm, whose sign is in accordance with the ECD sign of the PDPA (Figure 2e,f).^52^ Once chiroptical studies indicated that both *para*- and *meta*-substituted PPAs and PDPAs are well folded into a screw sense helical structure, AFM studies were performed to determine how their self-assembly into 2D nanostructures is affected by the absence (*para*-substituted) and the presence (*meta*-substituted) of facial asymmetry in the helical grooves. Thus, 5 mL of dilute solutions of *p*-poly­(*R*)-**1** and *m*-poly­(*R*)-**1** (PPAs) in CHCl_3_ was spin-coated onto freshly exfoliated highly oriented pyrolytic graphite (HOPG) and left under a solvent atmosphere for 2 h.^25–27^ HOPG is the substrate of choice to perform AFM studies based on all previous structural studies of helical polymers using AFM.^23–36^ The same protocol was employed for solutions of *p*-poly­(*S*)-**2** and *m*-poly­(*S*)-**2** (PDPAs) in DMF and CHCl_3_. High-resolution AFM images of *p-*poly­(*R*)-**1**/*m-*poly­(*R*)-**1** (PPAs) and *p*-poly­(*S*)-**2**/*m-*poly­(*S*)-**2** (PDPAs) were obtained, showing a direct relationship between the aromatic substitution patterns of PPAs and PDPAs and their self-assembly properties.

### *Para*-substituted PPA and PDPA

High-resolution images of *para-*substituted PPA [*p*-poly­(*R*)-**1**] (Figure 3a and Figure S6) and PDPA [*p*-poly­(*S*)-**2**] (Figure 3c and Figure S7) show the formation of 2D crystals consisting of well-ordered parallel stacks of polymer chains. From these images, it is possible to extract key structural parameters, such as the helical pitch or the orientation of the external helix, which match those previously reported.^34,37,41^ These 2D crystals usually appear in combination with superhelical fibers (Figure 3b) obtained by 3D packing of a bundle of helical polymers.

**Figure 3:**
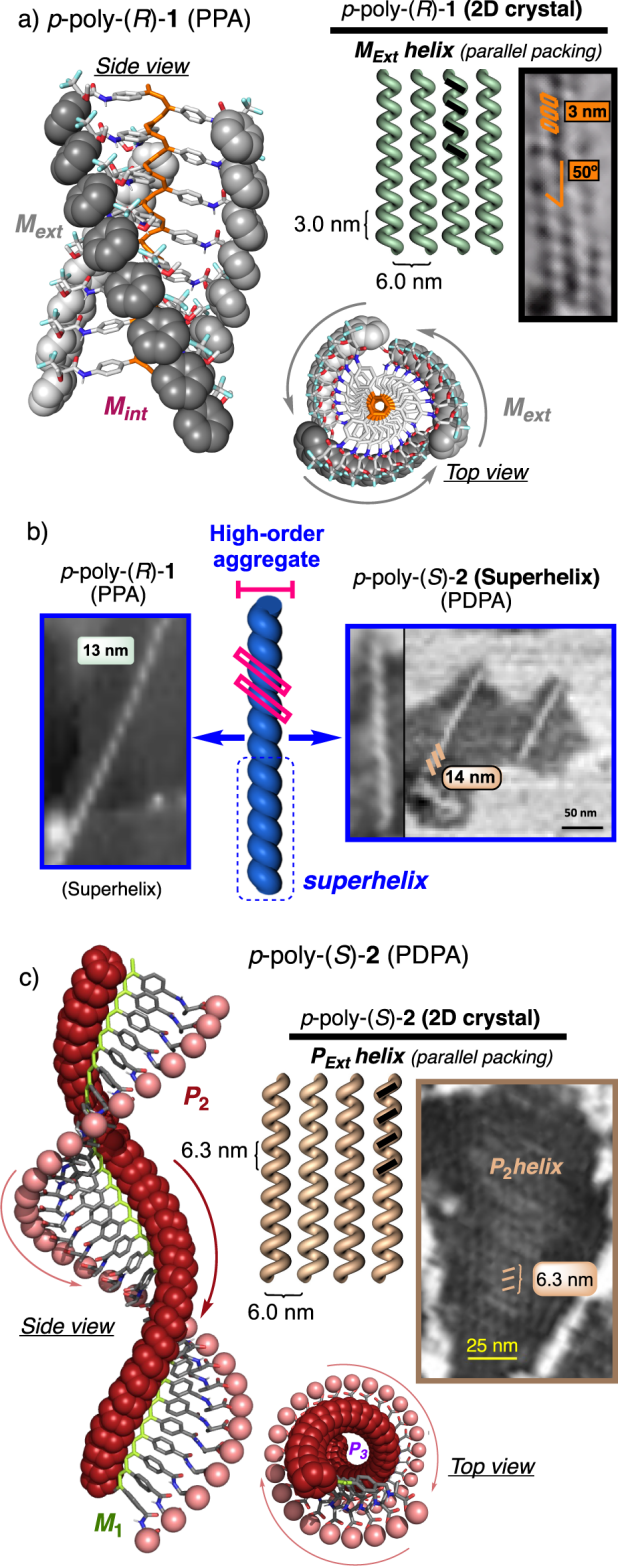
(a) Estimated secondary structures and AFM images of 2D crystals of *p-*poly­(*R*)-**1** (PPA) prepared in CHCl_3_. (b) AFM images showing superhelices of *p*-poly­(*R*)-**1** and *p*-poly­(*S*)-**2**. (c) Estimated secondary structures and AFM images of 2D crystals of *p-*poly­(*S*)-**1** (PDPA) prepared in DMF.

### *Meta*-substituted PPA and PDPA

*m*-Poly­(*R*)-**1** self-assembles into 2D toroidal nanostructures, which are observed among classical 2D crystals or superhelices [Figure 4a and Figures S8–S11 for Archimedean nanospirals, Figure 4a and Figures S12–S13 for superhelices, and Figure 4a and Figures S14–S16 for 2D crystals], while *m*-poly­(*S*)-**2** self-assembles only into 2D toroidal nanostructures (Figure 5 and Figure S17).

**Figure 4:**
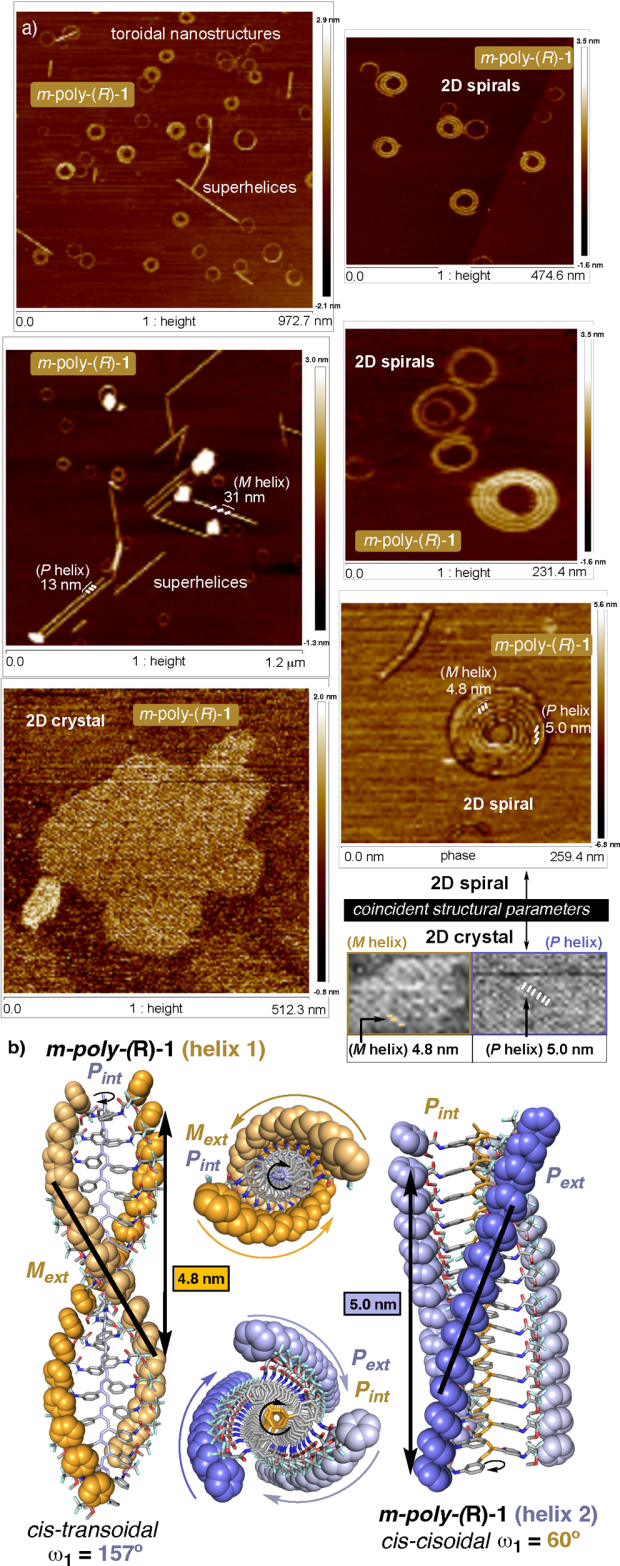
(a) AFM images and (b) approximate secondary structures built for *m*-poly­(*R*)-**1** (PPA) by combining information from AFM and ECD.

**Figure 5:**
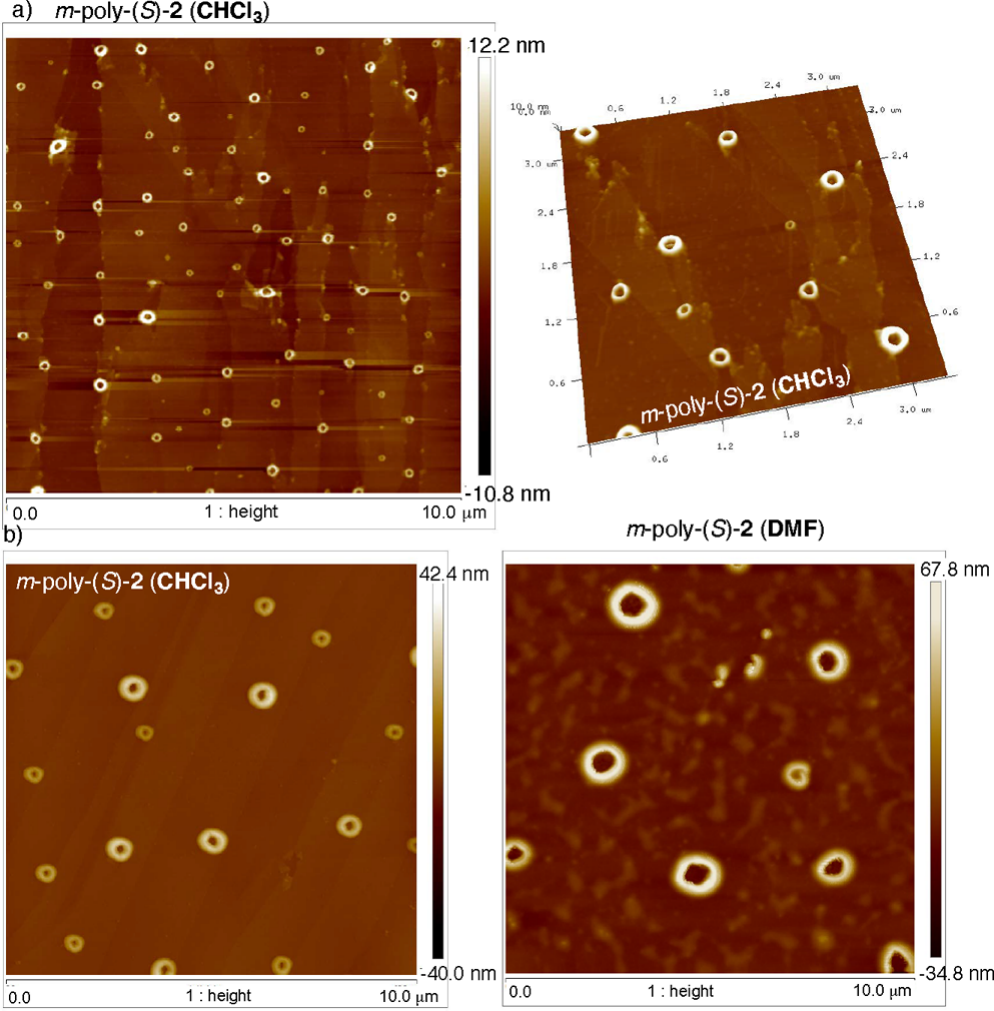
AFM images of poly­(*S*)-**2** in (a) CHCl_3_ and (b) DMF deposited on HOPG.

Remarkably, in the case of *m*-poly­(*R*)-**1** (PPA), high-resolution images of these concentric toroidal nanostructures allow one to decipher their real framework, which consists of planar Archimedean nanospirals obtained from the self-assembly of a single helical polymer chain (Figure 4a and Figures S8–S11). The helical parameters (pitch, width, and packing distance) in both the 2D crystal (Figure 4a and Figures S14–S16) and the planar Archimedean nanospirals (Figure 4a and Figures S8–S11) are almost coincident (helix pitch, helix width, and packing distance), indicating that the 2D Archimedean nanospirals are generated by intrachain self-assembly of a single polymer chain.

Thus, *m*-poly­(*R*)-**1** (polymer sequence, primary structure) folds into a *P* helix (secondary structure) that self-assembles into an Archimedean nanospiral (tertiary structure) (Figure 4a). Fascinatingly, both 2D crystals and 2D Archimedean nanospirals show the presence of two different helical scaffolds, which are coincident with those obtained in previous structural studies based on photochemical electrocyclization of the polyene backbone.^37^ Thus, in some regions of the AFM images, a *P* helix with a helical pitch of ca. 5.0 nm is observed, whereas in other regions, an *M* helix with a pitch of ca. 4.8 nm is found (Figure 4a). These two helices are consistent with the presence of a *cis–cisoidal* helix (ω_1_ ca. 60°, *P* internal and *P* external, Figure 4b) and a *cis–transoidal* helix (ω_1_ ca. 150°, *P* internal and *M* external, Figure 4b), as previously elucidated by photochemical electrocyclization studies.^37^ Moreover, in addition to these helices, *P*- and *M*-oriented superhelices were also observed, with helical pitches of 13 and 31 nm, respectively (Figure 4a). These superhelices are formed by self-assembly of several individual helices, which depending on their number, superhelices with different parameters (width, pitch, and screw sense) are obtained.

In the case of *m*-poly­(*S*)-**2** (PDPA), toroidal nanostructures are observed covering the HOPG AFM substrate after spin-coating diluted solutions of *m*-poly­(*S*)-**2** in DMF or CHCl_3_ (Figure 5). In this case, the height of the nanotoroids (ca. 22 nm; see Figure S17) indicates that there is an additional process of parallel self-assembly of toroidal nanostructures, which precludes the observation of individual helices. These toroidal nanostructures were observed by preparing samples in both DMF and CHCl_3_ solvents, indicating the high tendency of this material to aggregate into these toroidal nanosystems.

## Conclusions

In conclusion, it was demonstrated by different examples that changes in the aromatic substitution pattern of chiral *s*ubstituted PPAs and PDPAs result in changes not only in the dynamic helical behavior of the helices but also in their self-assembly properties. Thus, while in *para*-substituted PPAs or PDPAs, the helices possess symmetric grooves due to the location of the pendant group in the middle of the aryl ring attached to the polyene backbone, i.e., *para-*substitution, in the case of *meta-*substituted PPAs or PDPAs, a facial asymmetry is introduced into the helical grooves due to the location of the pendant in a nonsymmetrical position at the aryl ring of the phenylacetylene backbone, i.e., *meta-*substitution.

As a result, the presence of hydrophobic/hydrophilic facial asymmetry in the helical grooves of *meta*-substituted PPAs or PDPAs results in different self-assembly properties compared to their *para*-substituted counterparts. Therefore, while in *para*-substituted PPAs or PDPAs, 2D crystals are formed by parallel self-assembly of polymer chains, in the case of *meta*-substituted PPAs or PDPAs, single-chain 2D Archimedean spiral nanostructures or nanotoroidal structures are formed through intramolecular self-assembly. This finding indicates that the facial asymmetry at the helical grooves in *meta*-substituted PPAs or PDAs plays an important role in the formation of these interesting single-chain 2D Archimedean spiral nanostructures.

Thus, we present an approach to obtain 2D Archimedean spirals by molecular self-assembly, in addition to those based on amphiphilic macromolecules (block copolymers) or discrete molecules. These studies show how macromolecular self-assembly can be used to prepare higher hierarchical level nanostructures that attempt to mimic sophisticated frameworks similar to those found in living systems.

## Materials and Methods

CD measurements were done in a Jasco-720. The concentrations of polymer used for CD measurements were 0.9 or 1.6 mM for PPAs and PDPAs, respectively, in the corresponding solvent.

UV spectra were registered in a Jasco V-630. The concentrations of polymer used for UV measurements were 0.9 or 1.6 mM for PPAs and PDPAs, respectively, in the corresponding solvent.

GPC studies were carried out in a Waters Alliance instrument equipped with Phenomenex GPC columns (THF, flow = 1 mL/min). The amount of polymer used for GPC measurements was 0.5 mg/mL.

Circularly polarized luminescence (CPL) and emission measurements were performed by using an in-house-developed JASCO CPL spectrofluoropolarimeter. The samples were excited using a 90° geometry with a green InGaN (3 mm, 2 V) LED source (Luckylight Electronics Co., LTD, λ_max_ = 517 nm, HWHM = 15 nm). The following parameters were used: emission slit width ≈ 10 nm, integration time = 4 s, scan speed = 50 nm/min, three accumulations.

PL quantum yields were measured by using an Edinburgh Spectrofluorometer FS5 equipped with an integrating sphere.

## Supplementary Material



## Notes

Materials and methods, synthesis and characterization of monomers, synthesis and characterization of polymers, computational details, and supporting references (PDF)
