# Supplementary material for: Facial Asymmetry in the Helical Grooves of Chiral Helical Polymers to Create 2D Single-Chain Archimedean Spiral Nanostructures
Source: ACS Nano. 2025 Sep 10;19(37):33423–9. doi: 10.1021/acsnano.5c10476 (PMC12462248; doi:10.1021/acsnano.5c10476)
Supplement: Supplementary file 1 [file nn5c10476_si_001.pdf]

Facial Asymmetry in the Helical Grooves of Chiral Helical Polymers to Create 2D-Single Chain Archimedean Spiral Nanostructures

Juan José Tarrío,[†b] Francisco Rey-Tarrío,[†b] Borja Hermida,[b] Berta Fernández,[c] Jeanne Crassous,[d] Emilio Quiñoá,[b] Rafael Rodríguez,[a*] and Félix Freire[a*]

[a]*CINBIO and Departamento de Química Orgánica. Campus Lagoas-Marcosende, Universidade de Vigo, Vigo, E-36310, Spain.*

[b]*Centro Singular de investigación en Química Biolóxica e Materiais Moleculares (CiQUS) and Departamento de Química Orgánica, Universidade de Santiago de Compostela, E-15782 Santiago de Compostela, Spain.*

[c]*Departamento de Química Física, Universidade de Santiago de Compostela, E-15782 Santiago de Compostela, Spain.*

[d]*Univ Rennes, CNRS, ISCR (Institut des Sciences Chimiques de Rennes) – UMR 6226, F-35000 Rennes, France.*

Table of Contents

Materials and Methods....................3
Synthesis of monomers....................4
Synthesis of Polymers....................8
  Polymerization....................8
  Post-polymerization Coupling....................11
  GPC data....................12
Additional ECD experiments....................13
Additional AFM Images....................14
Computational Details....................25
Supporting References....................26

Materials and Methods

CD measurements were done in a Jasco-720. The concentration of polymer used for CD measurements were 0.9 or 1.6 mM for PPAs and PDPAs, respectively, in the corresponding solvent.

UV spectra were registered in a Jasco V-630. The concentration of polymer used for UV measurements were 0.9 or 1.6 mM for PPAs and PDPAs, respectively, in the corresponding solvent.

GPC studies were carried out in a Waters Alliance equipped with Phenomenex GPC columns (THF, flow = 1 mL/min). The amount of polymer used for GPC measurements was 0.5 mg/mL.

Circularly polarized luminescence (CPL) and emission measurements were performed using an in-house-developed JASCO CPL spectrofluoropolarimeter. The samples were excited using a 90°-geometry with a green InGaN (3 mm, 2 V) LED source (Luckylight Electronics Co., LTD, λ_{max} = 517 nm, HWHM = 15 nm). The following parameters were used: emission slit width ≈ 10 nm, integration time = 4 sec, scan speed = 50 nm/min, 3 accumulations.

PL Quantum yields were measured using an Edinburgh Spectrofluorometer FS5 equipped with an integrating sphere.

Synthesis of monomers

Perfluorophenyl 3-iodobenzoate

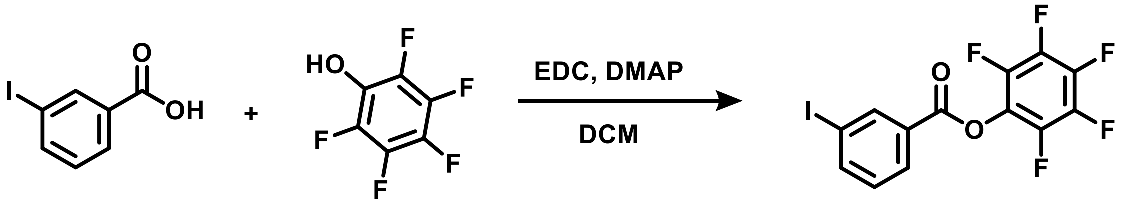

1 2 3

3-Iodobenzoic acid (**1**, 1.48 g, 5.97 mmol), EDC (1.19 g, 6.24 mmol), DMAP (0.073 g, 0.6 mmol) and 50 mL dry DCM were added into a 250 mL flask. After stirring for 15 min to activate the acid, pentafluorophenol (**2**, 1.00 g, 5.43 mmol) was added. The mixture was stirred at rt for 12 h and a white precipitate was formed. Then, the solid was filtrated and washed with hexane. After concentration in a rotary evaporator, the crude product was purified by silica gel column chromatography (40-63 mesh) using a mixture of hexane and ethyl acetate (95:5 by volume) as eluent. After evaporation of solvents in a rotary evaporator, a white solid was obtained in 76% yield.

^{1}H NMR (300MHz, $CDCl_3$) δ (ppm): 8.52 (1H, s), 8.16 (1H, d), 8.01 (1H, d), 7.28 (1H, t)

^{19}F NMR (282.3 MHz, $CHCl_3$) δ (ppm): -152.4, -157.7, -162.2

^{13}C NMR (75MHz, $CDCl_3$) δ (ppm): 161.1, 143.4, 141.3, 139.3, 138.0, 136.3, 130.4, 129.7, 128.7, 125.2, 94.0.

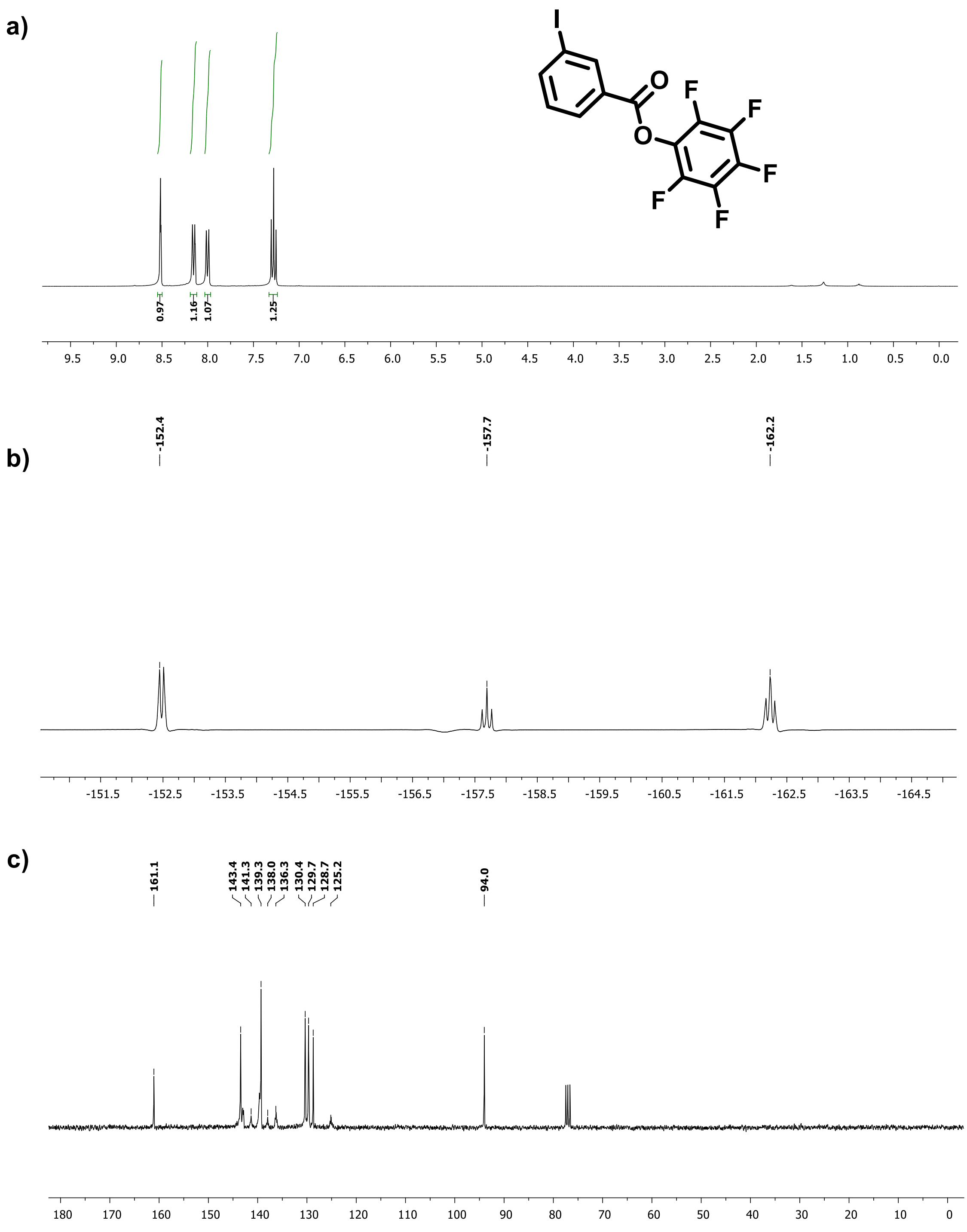

Figure S1. a) ^{1}H, b) ^{19}F and c) ^{13}C NMR spectra of **3** (perfluorophenyl 3-iodobenzoate).

Perfluorophenyl 3-(phenylethynyl)benzoate

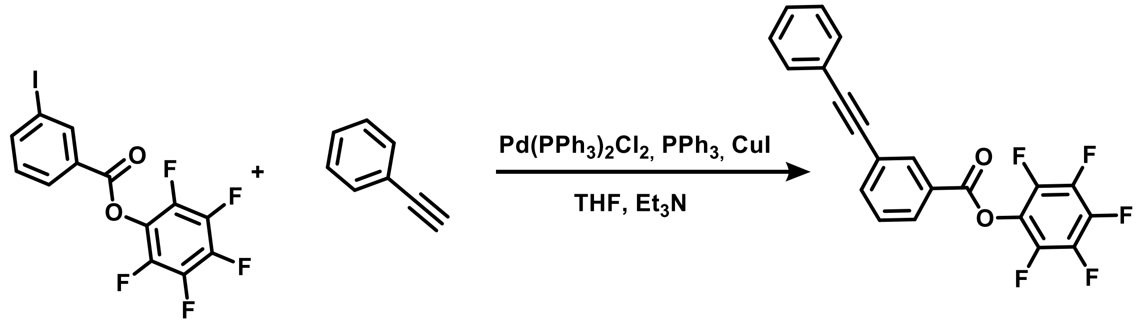

3 4 *m*-**M-1**

Perfluorophenyl 3-iodobenzoate (**3**, 1.7 g, 4.1 mmol), $PdCl_2(PPh_3)_2$ (143 mg, 0.205 mmol), CuI (78 mg, 0.41 mmol), PPh_3 (107 mg, 0.41 mmol) were added to 20 mL of THF and 2.7 mL of Et_3N (20.5 mmol) under nitrogen. After the catalysts was completely dissolved, phenylacetylene (0.67 mL, 6.15 mmol) was injected into the flask and the mixture was stirred at rt for 24 h. The solid was removed by filtration and washed with diethyl ether. The filtrate was then concentrated in a rotary evaporator. The crude product was purified by silica gel column chromatography (40-63 mesh) using a mixture of hexane and ethyl acetate (95:5 by volume) as eluent. *m*-**M-1** was obtained as a white solid in 67% yield.

1H NMR (300MHz, $CDCl_3$) δ (ppm): 8.39 (1H, s), 8.15 (1H, d), 7.85 (1H, d), 7.60 (2H, m), 7.53 (1H, t), 7.39 (3H, m).

^{19}F NMR (282.3 MHz, $CHCl_3$) δ (ppm): -152.4, -157.8, -162.3

^{13}C NMR (75 MHz, $CDCl_3$) δ (ppm): 162.0, 137.3, 133.6, 131.7, 130.0, 129.0, 128.8, 128.4, 127.3, 124.5, 122.6, 91.1, 87.6

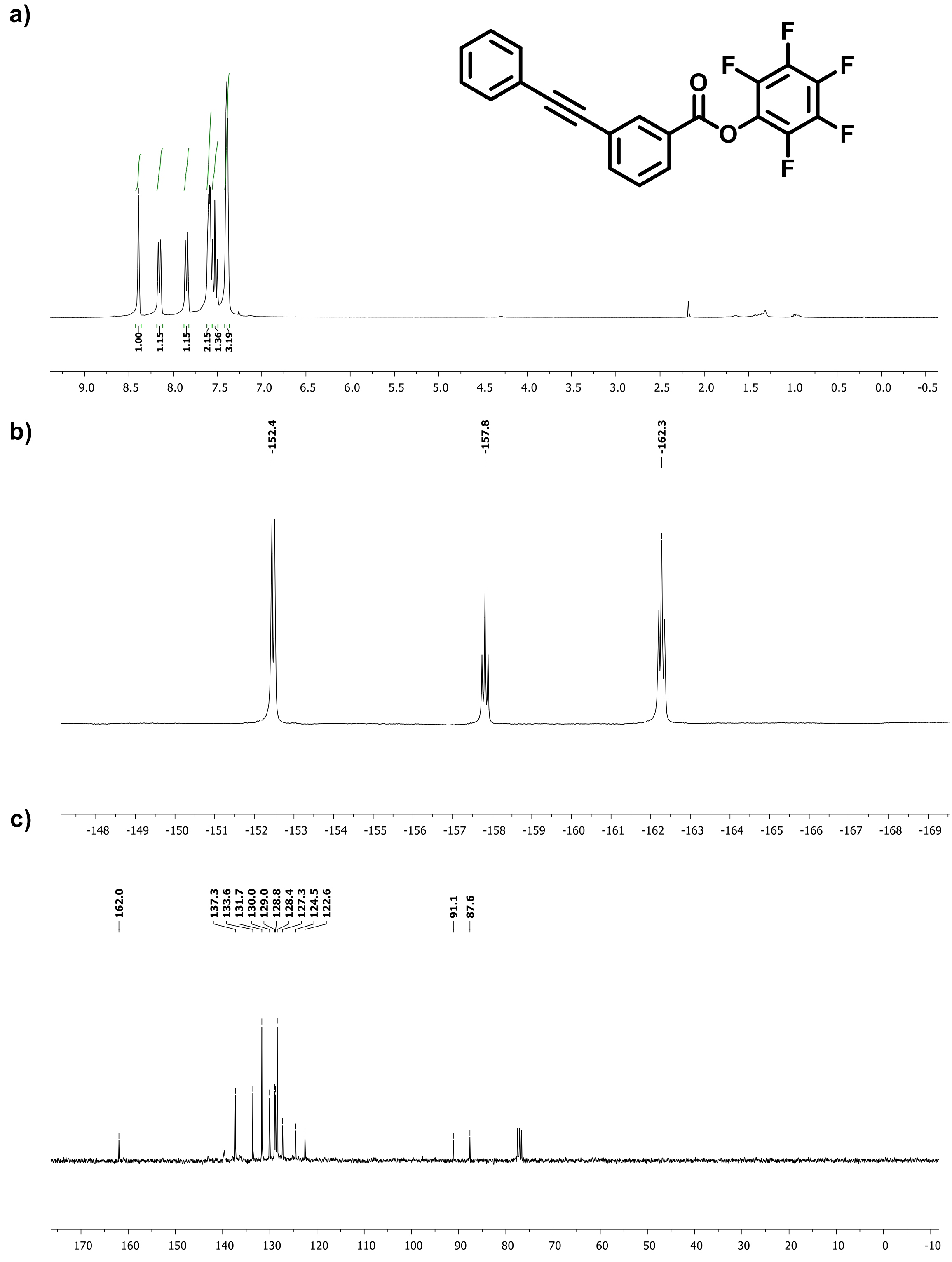

Figure S2. a) ^{1}H, b) ^{19}F and c) ^{13}C NMR spectra of *m*-M-**1** ($CDCl_3$).

Synthesis of Polymers

p-Poly-(*R*)-**1**, *m*-poly-(*R*)-**1** and *p*-poly-(*S*)-**2** have been prepared according to our previously reported methods.[S1-S3] The preparation of *m*-poly-(*S*)-**2** is detailed below.

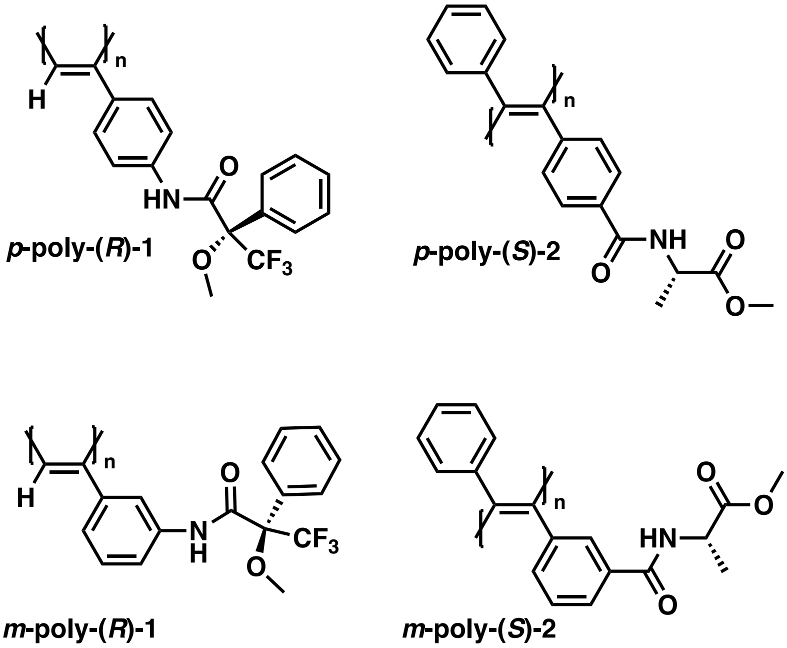

Figure S3: Structure of the polymers used in this work.

Polymerization

Polymerization of *m*-poly-(*R*)-**1** was previously reported.[S3] The polymerization reaction was carried out under a nitrogen atmosphere using standard Schlenk technique, unless otherwise specified. A typical procedure for the polymerization of *m*-poly-PF_6 is given below as an example following the conditions of reference **S2**.

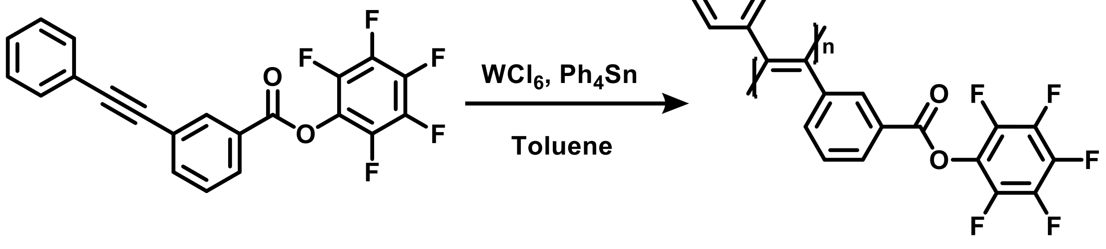

m-**M-1** *m*-**Poly-1**

20.4 mg of WCl_6 and 44.0 mg of Ph_4Sn were added into a baked 10 mL Schlenk tube with a stopcock in the side arm. The tube was evacuated under vacuum and then flushed with dry nitrogen three times through the side arm. Freshly distilled toluene (1 mL) was injected into the tube to dissolve the mixture of catalysts and then, the catalyst solution was aged at 100 °C for 10 min. The monomer solution was prepared in another tube by dissolving 200 mg of *m*-**M-1** in 1.6 mL of toluene

and was transferred to the catalyst solution using a hypodermic syringe. The reaction mixture was stirred at 100 °C for 24 h. The solution was cooled to rt, diluted with CH_2Cl_2 and then precipitated in a large amount of methanol. The precipitate was allowed to stand overnight and then collected by filtration. The polymer was washed with methanol and hexane and dried under vacuum at room temperature to a constant weight. *m*-Poly-**1** was obtained as a brown solid in 71% yield.

^{1}H-NMR (300MHz, $CDCl_3$), δ (TMS, ppm): 7.70-7.30, 7.06-6.05, 6.05-5.55.

^{19}F NMR (282.3MHz, $CDCl_3$), δ (TMS, ppm): -153.4, -157.6, -162.6.

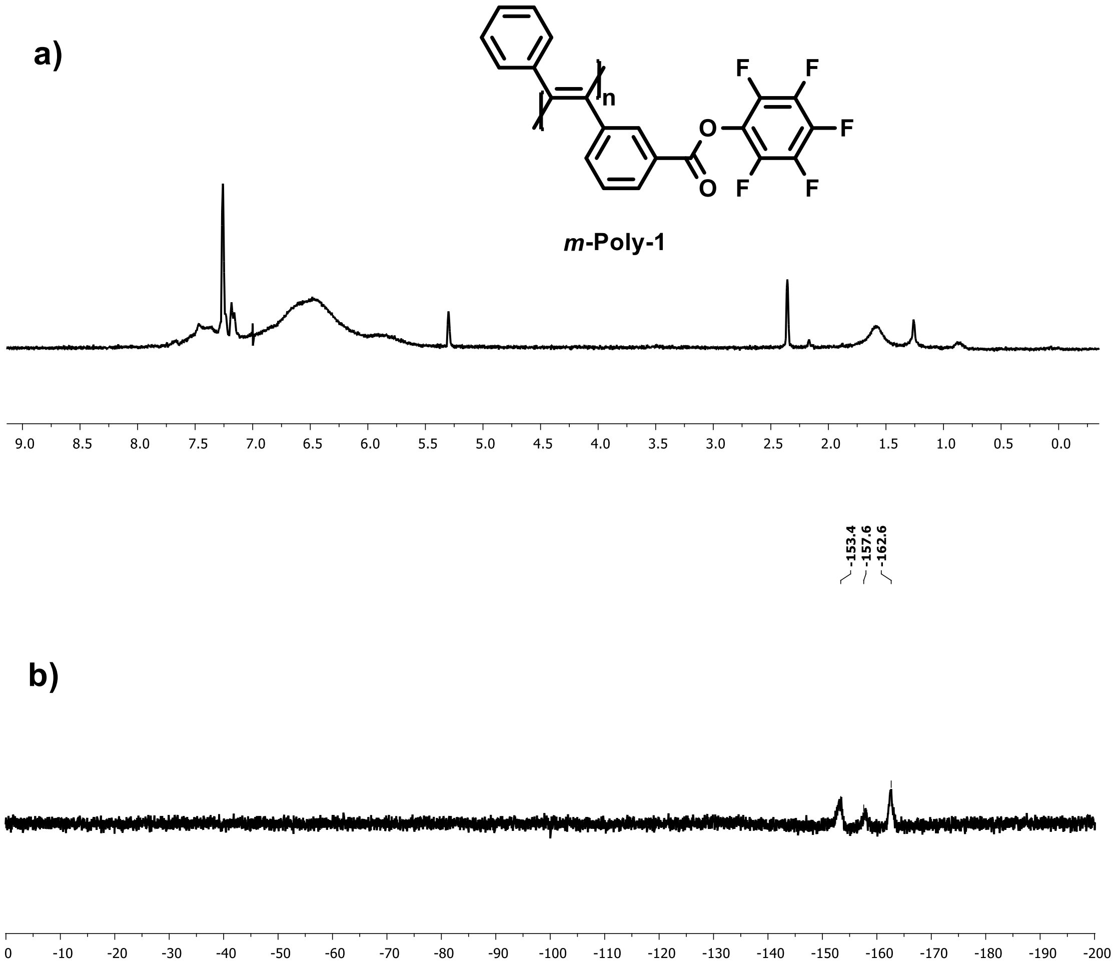

Figure S3. a) ^{1}H-NMR and b) ^{19}F-NMR spectra of *m*-Poly-**1**.

Post-polymerization Coupling

The post-polymerization reaction was carried out using standard peptide coupling conditions. A typical procedure for the coupling of alanine methyl ester is given below as an example following the conditions of Ref. **S1**.

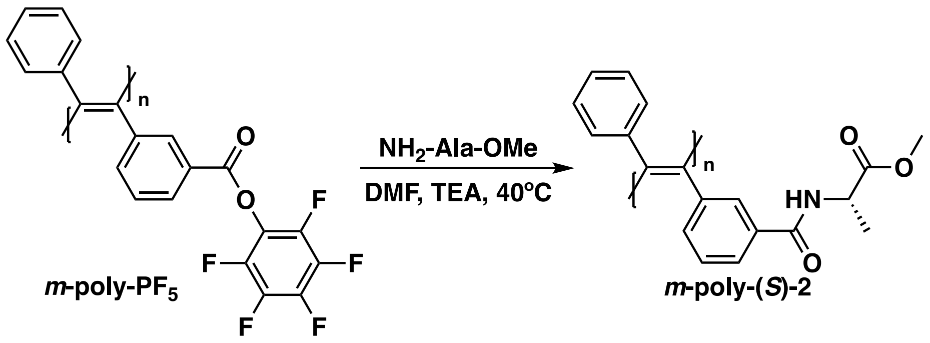

20 mg (0.052 mmol) of *m*-Poly-**1**, 8.6 mg (0.062 mmol) of *L*-alanine methyl ester hydrochloride and 2 mL dry DMF with a drop of TEA were added into a 10 mL reaction tube with magnetic stirring bar under nitrogen. The reaction solution was stirred for 12 h at 40 °C. Next, the solution mixture was added dropwise to 300 mL methanol through a cotton filter under vigilant stirring. The precipitate was kept still overnight and then filtered. The obtained polymer was washed with water and hexane several times and dried at rt to a constant weight. *m*-poly-(*S*)-**2** was obtained as a yellow solid in 89 % yield.

^{1}H NMR (300 MHz, $CDCl_3$), δ (TMS, ppm): 7.73, 6.92, 6.48, 5.98, 4.59, 3.74, 1.42.

^{19}F NMR (282.3 MHz, $CDCl_3$), δ (TMS, ppm): no signals.

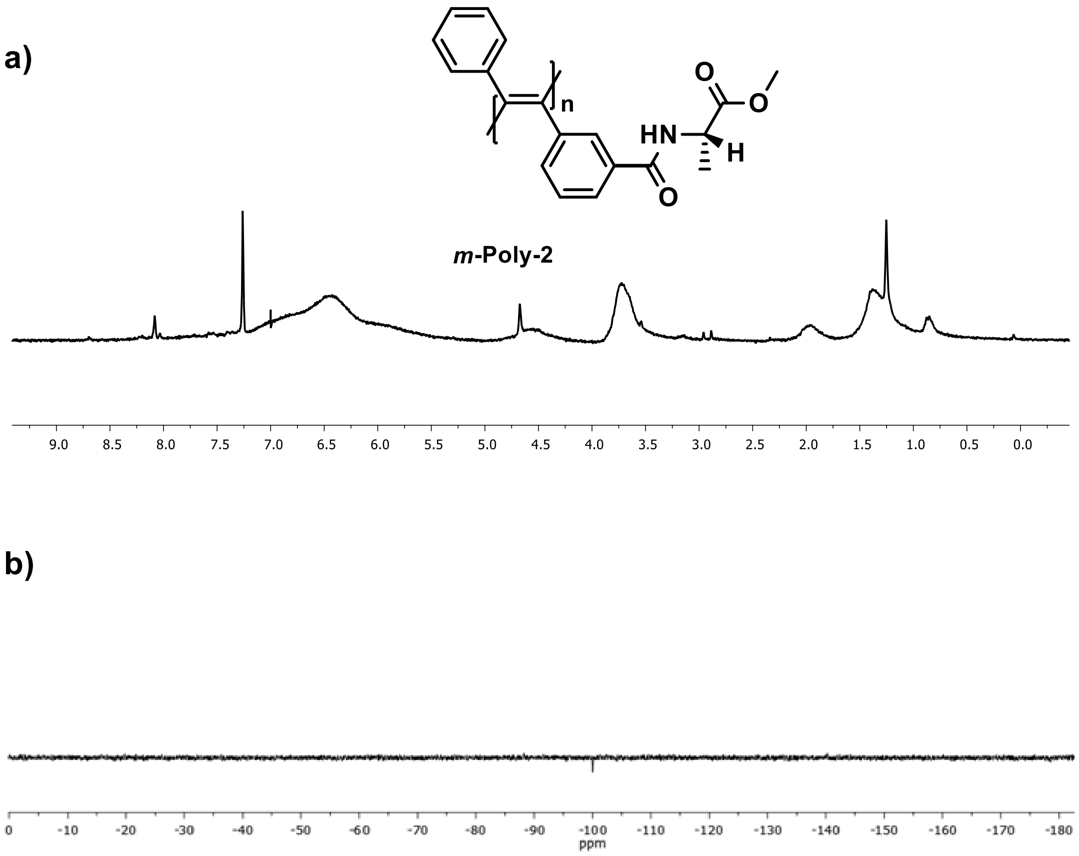

Figure S4. a) ^{1}H-NMR and b) ^{19}F-NMR spectra of *m*-Poly-**2**.

GPC data

Table 1. GPC polymer data.

Polymer	M_n	M_w	*Đ*
p-poly-(*R*)-**2**	$5.02 \cdot 10^4$	$5.19 \cdot 10^4$	1.03
m-poly-(*R*)-**2**	$2.65 \cdot 10^5$	$2.71 \cdot 10^5$	1.02
p-poly-PF_5	$1.40 \cdot 10^4$	$2.00 \cdot 10^5$	1.43
m-poly-PF_5	$1.50 \cdot 10^4$	$2.25 \cdot 10^4$	1.50

Additional ECD experiments

Additional ECD and UV-Vis spectra of PPAs (*p*-poly-(*R*)-**1** and *m*-poly-(*R*)-**1**) and PDPAs (*p*-poly-(*S*)-**2** and *m*-poly-(*S*)-**2**) in different solvents.

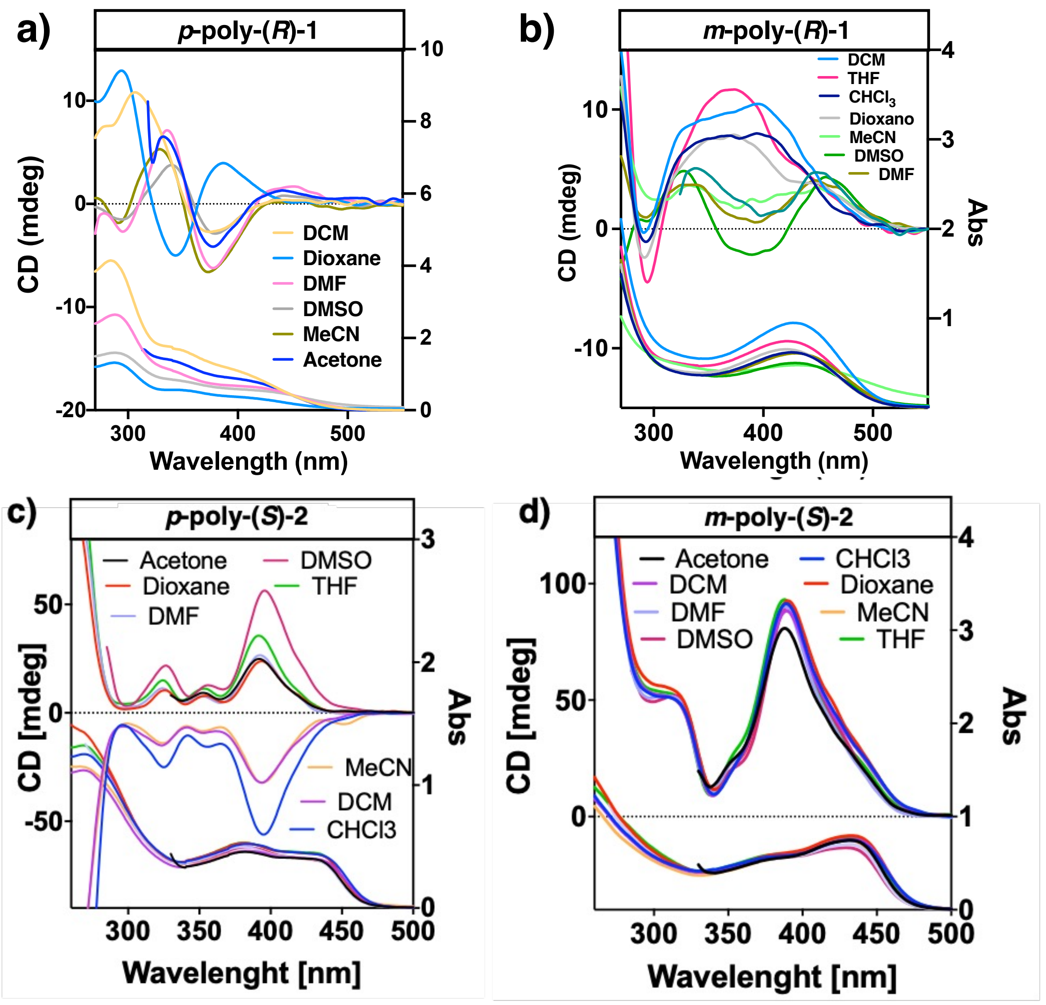

Figure S5. ECD and UV-Vis experiments in different solvents for (a) *p*-poly-(*R*)-**1**, (b) *m*-poly-(*R*)-**1**, (c) *p*-poly-(*S*)-**2** and (d) *m*-poly-(*S*)-**2** (PPAs, c= 0.9 mM; PDPAs, c= 1.6 mM).

Additional AFM Images

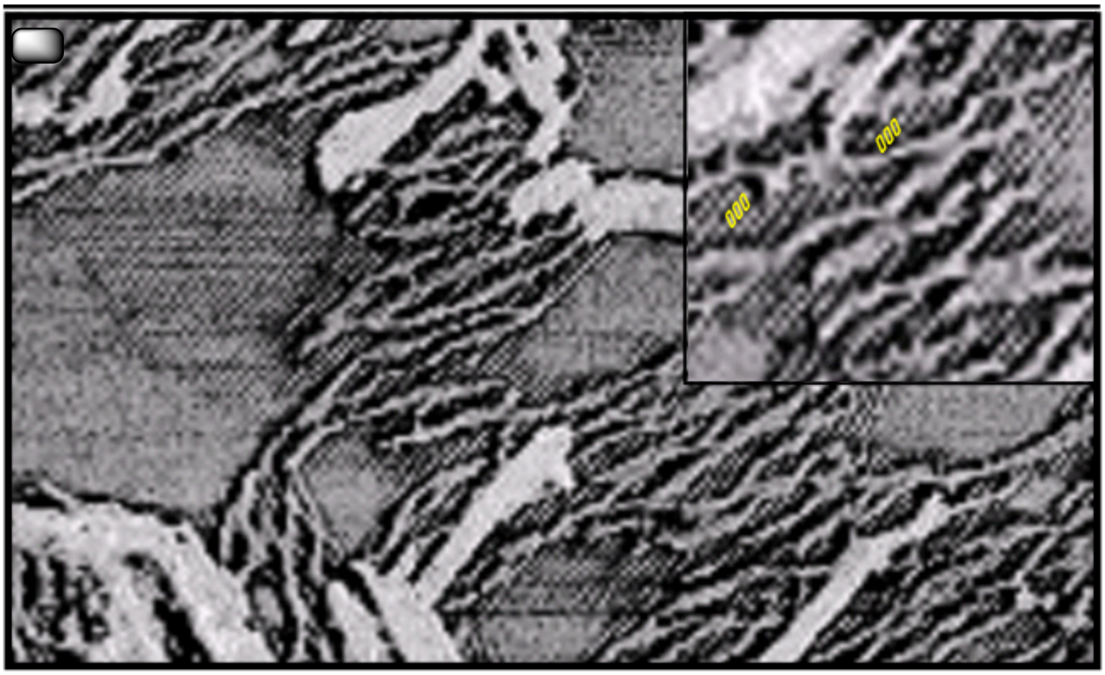

Figure S6. High resolution AFM images and of 2D-crystals generated by spin-coating a *p*-poly-(*R*)-1 $CHCl_3$ solution and using HOPG as substrate.

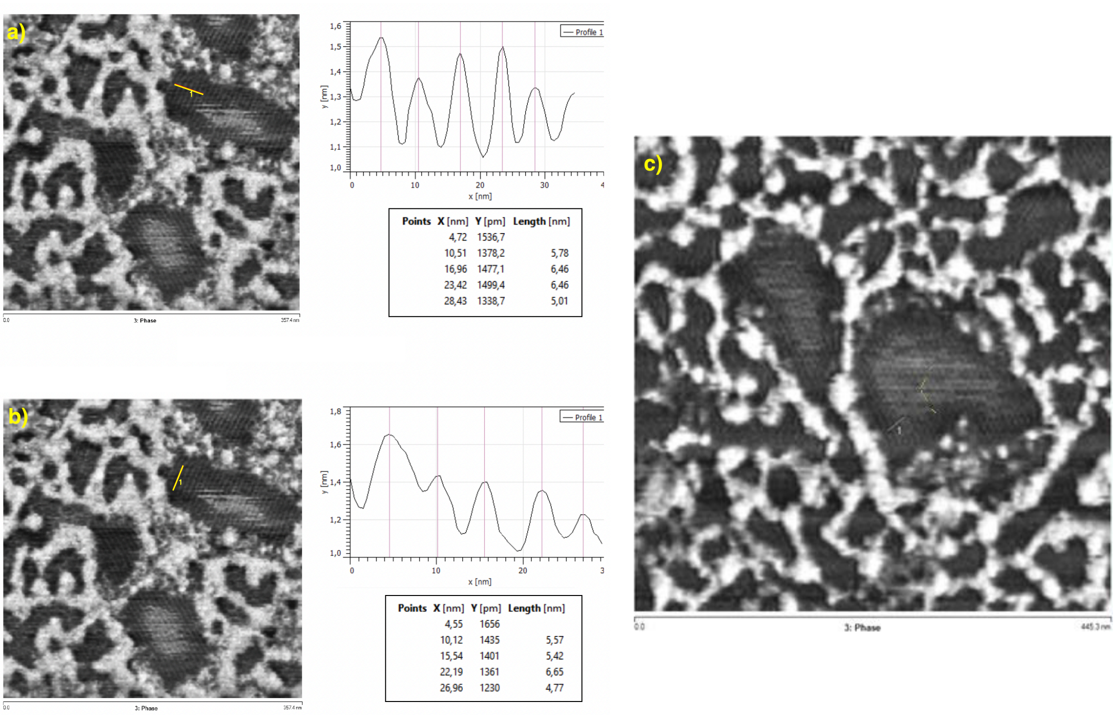

Points	X [nm]	Y [pm]	Length [nm]
	4,72	1536,7	
	10,51	1378,2	5,78
	16,96	1477,1	6,46
	23,42	1499,4	6,46
	28,43	1338,7	5,01

Points	X [nm]	Y [pm]	Length [nm]
	4,55	1656	
	10,12	1435	5,57
	15,54	1401	5,42
	22,19	1361	6,65
	26,96	1230	4,77

Figure S7. (a) to (c) High resolution AFM images and corresponding height profiles (yellow lines) showing the helical pitch and chain separation in the 2D-crystal generated by spin-coating a *p*-poly-(*S*)-**2** DMF solution, using HOPG as substrate.

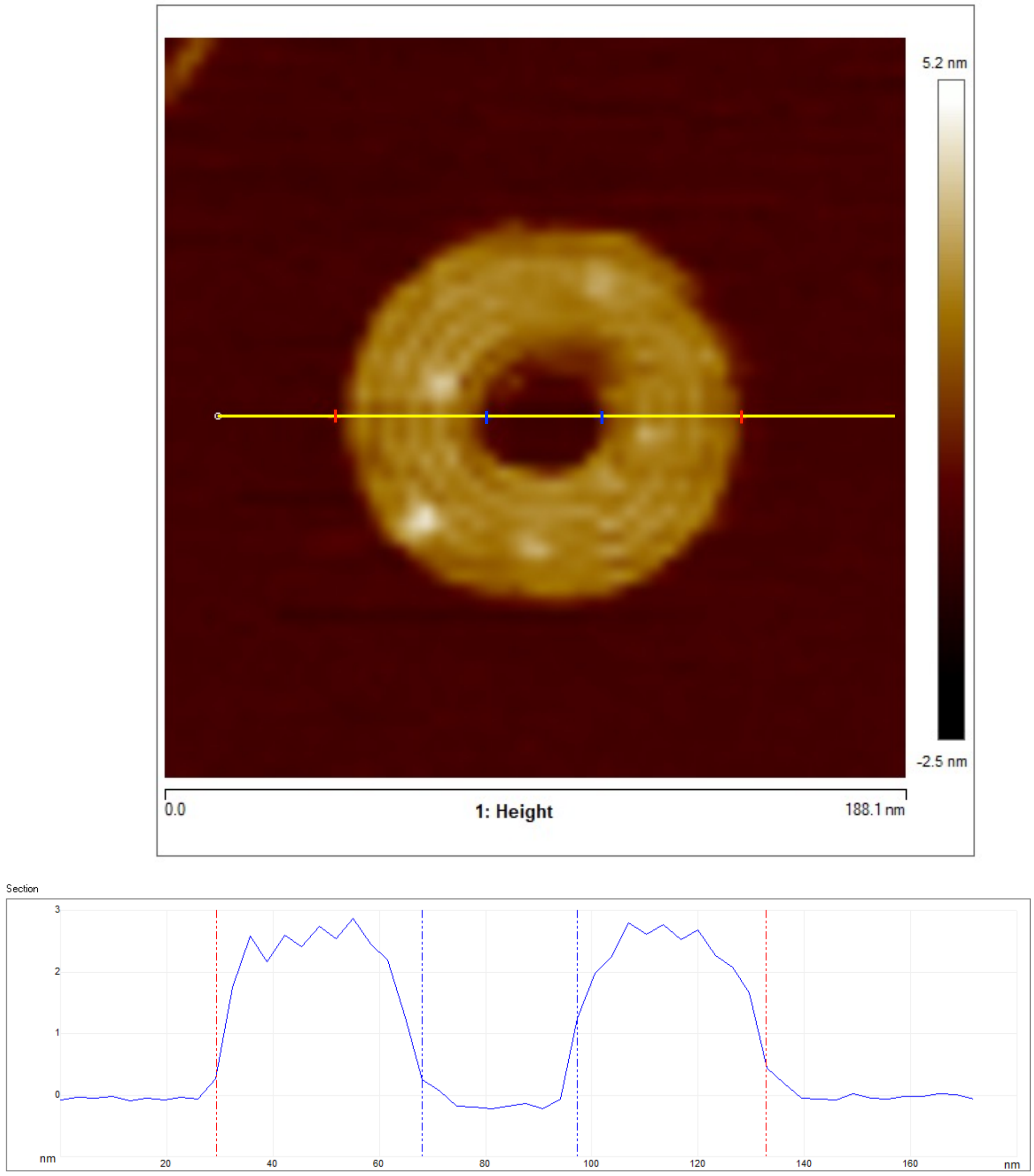

Pair	Horizontal Distance
1	29.332 (nm)
2	104.292 (nm)

Figure S8. High resolution AFM images and corresponding height profiles (yellow line) showing the helical height and width of the nanospiral generated by spin-coating an *m*-poly-(*R*)-1 $CHCl_3$ solution, using HOPG as substrate.

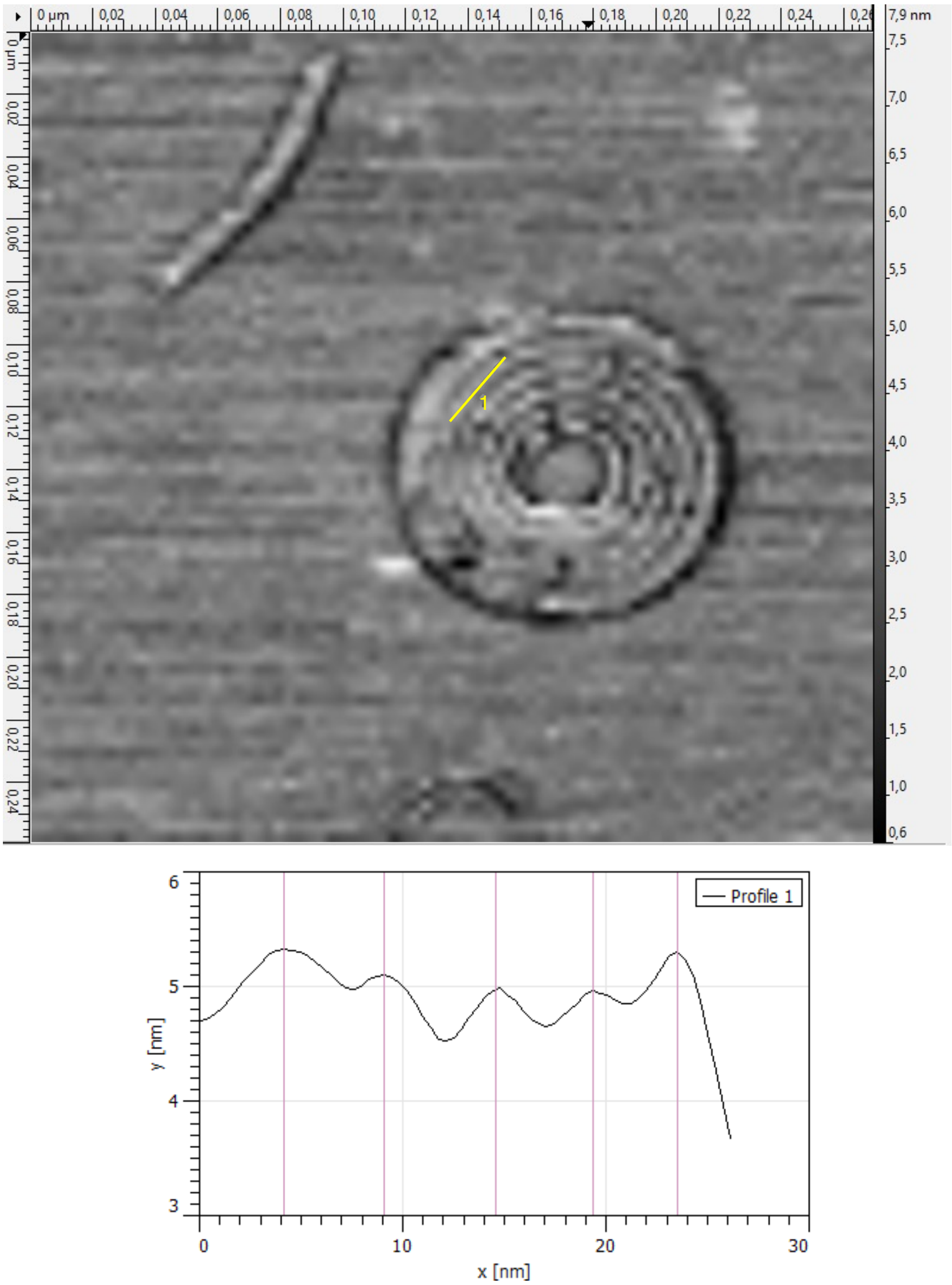

Points	X [nm]	Y [nm]	Length [nm]	Height [nm]	Angle [deg]
	4,19	5,337			
	9,09	5,110	4,91	-0,227	-2,65
	14,59	4,986	5,49	-0,124	-1,30
	19,34	4,971	4,76	-0,014	-0,17
	23,51	5,311	4,17	0,339	4,65

Figure S9. High resolution AFM images and corresponding height profile (yellow line) showing the helical pitch in the nanospiral generated by spin-coating an *m*-poly-(*R*)-1 $CHCl_3$ solution, using HOPG as substrate.

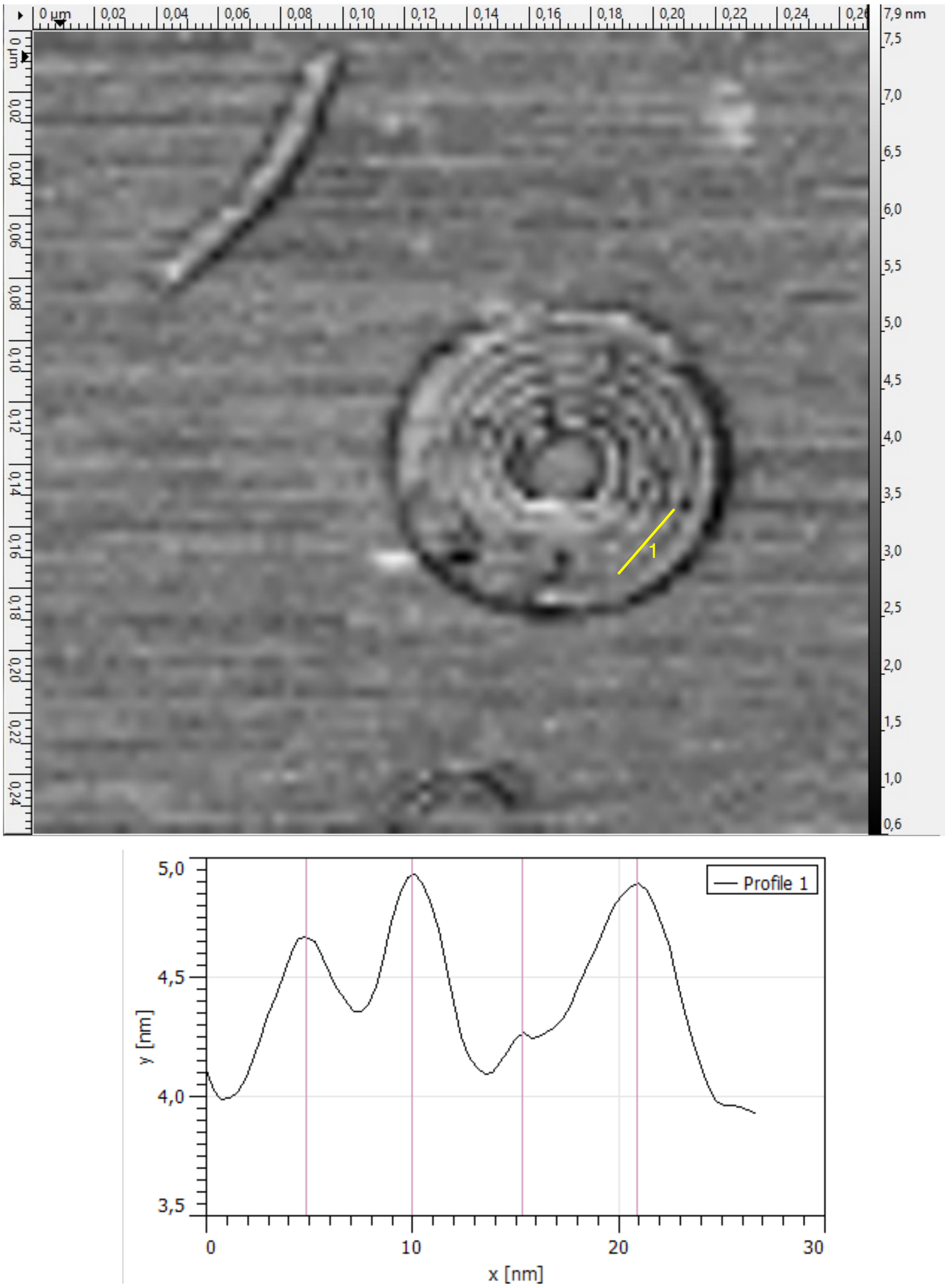

Points	X [nm]	Y [nm]	Length [nm]	Height [nm]	Angle [deg]
	4,83	4,674			
	10,01	4,933	5,18	0,259	2,87
	15,35	4,273	5,34	-0,660	-7,05
	20,89	4,894	5,54	0,622	6,40

Figure S10. High resolution AFM images and corresponding height profiles (yellow line) showing the helical pitch in the nanospiral generated by spin-coating an *m*-poly-(*R*)-1 $CHCl_3$ solution, using HOPG as substrate.

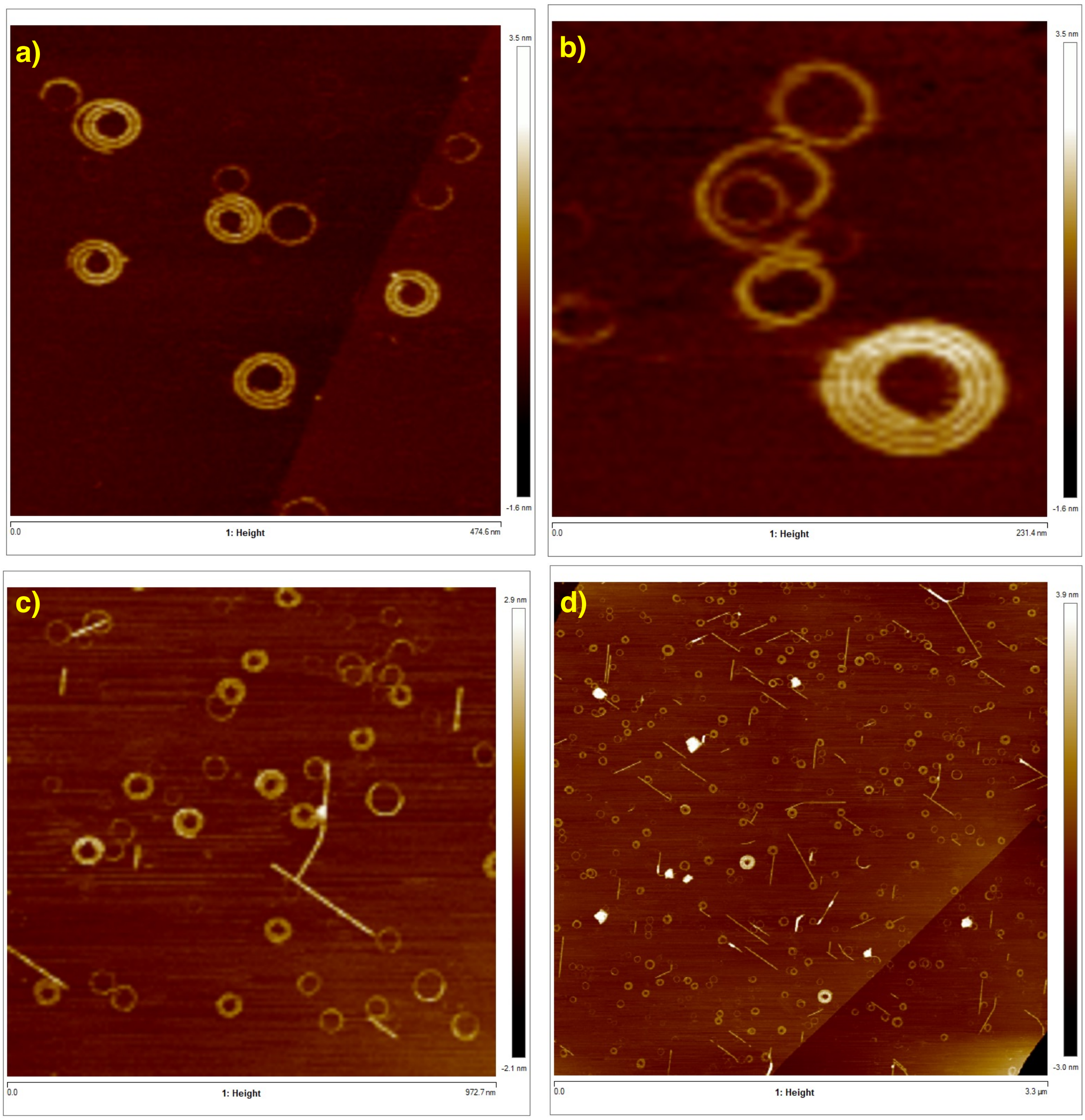

Figure S11. Large-scale high-resolution AFM images showing nanospirals and superhelices generated by spin-coating an *m*-poly-(*R*)-1 $CHCl_3$ solution and using HOPG as substrate.

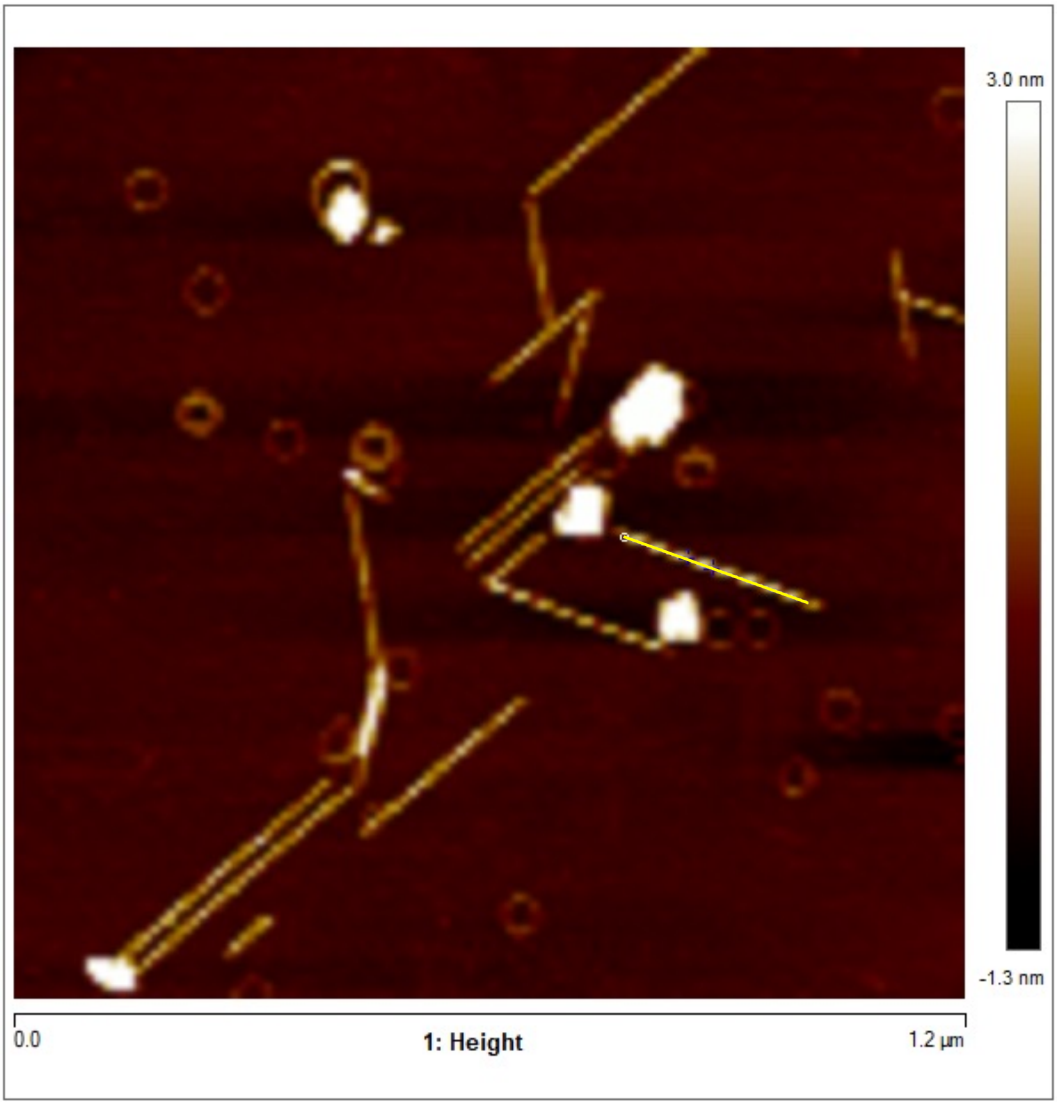

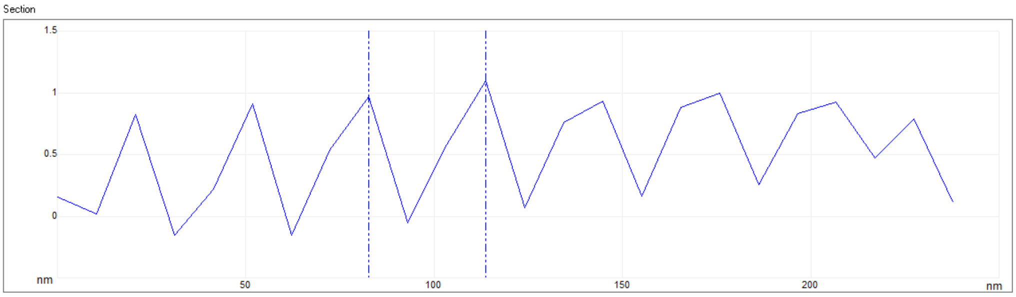

Pair	Horizontal Distance
1	31.079 (nm)

Figure S12. High resolution AFM images and corresponding height profiles (yellow line) showing the helical pitch in the superhelix generated by spin-coating an *m*-poly-(*R*)-1 $CHCl_3$ solution and using HOPG as substrate.

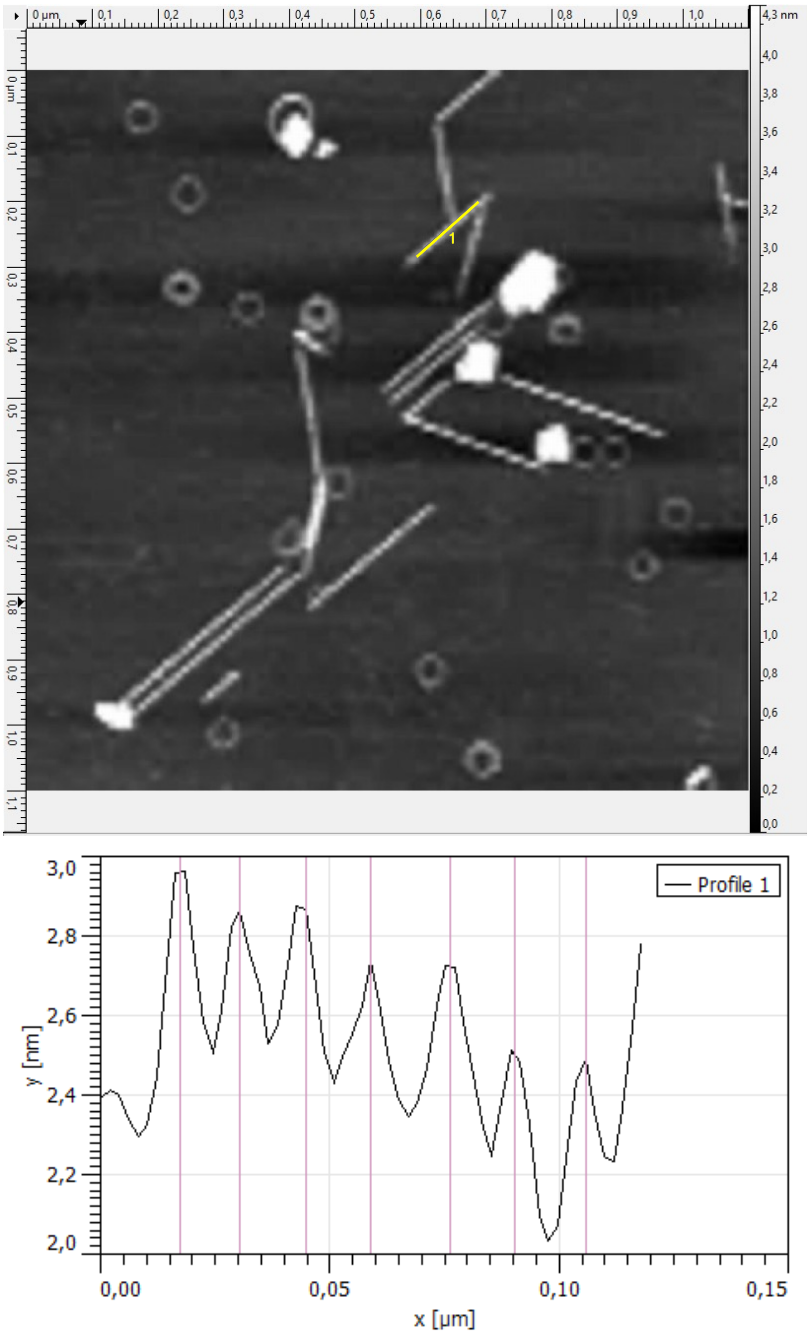

Points	X [nm]	Y [pm]	Length [nm]	Height [pm]	Angle [deg]
	17,3	2965			
	30,5	2865	13,3	-100	-0,43
	44,8	2869	14,2	5	0,02
	59,0	2740	14,2	-129	-0,52
	76,1	2728	17,1	-12	-0,04
	90,4	2504	14,3	-224	-0,90
	105,8	2495	15,3	-9	-0,03

Figure S13. High resolution AFM images and corresponding height profiles (yellow line) showing the helical pitch in the superhelix generated by spin-coating an *m*-poly-(*R*)-1 $CHCl_3$ solution and using HOPG as substrate.

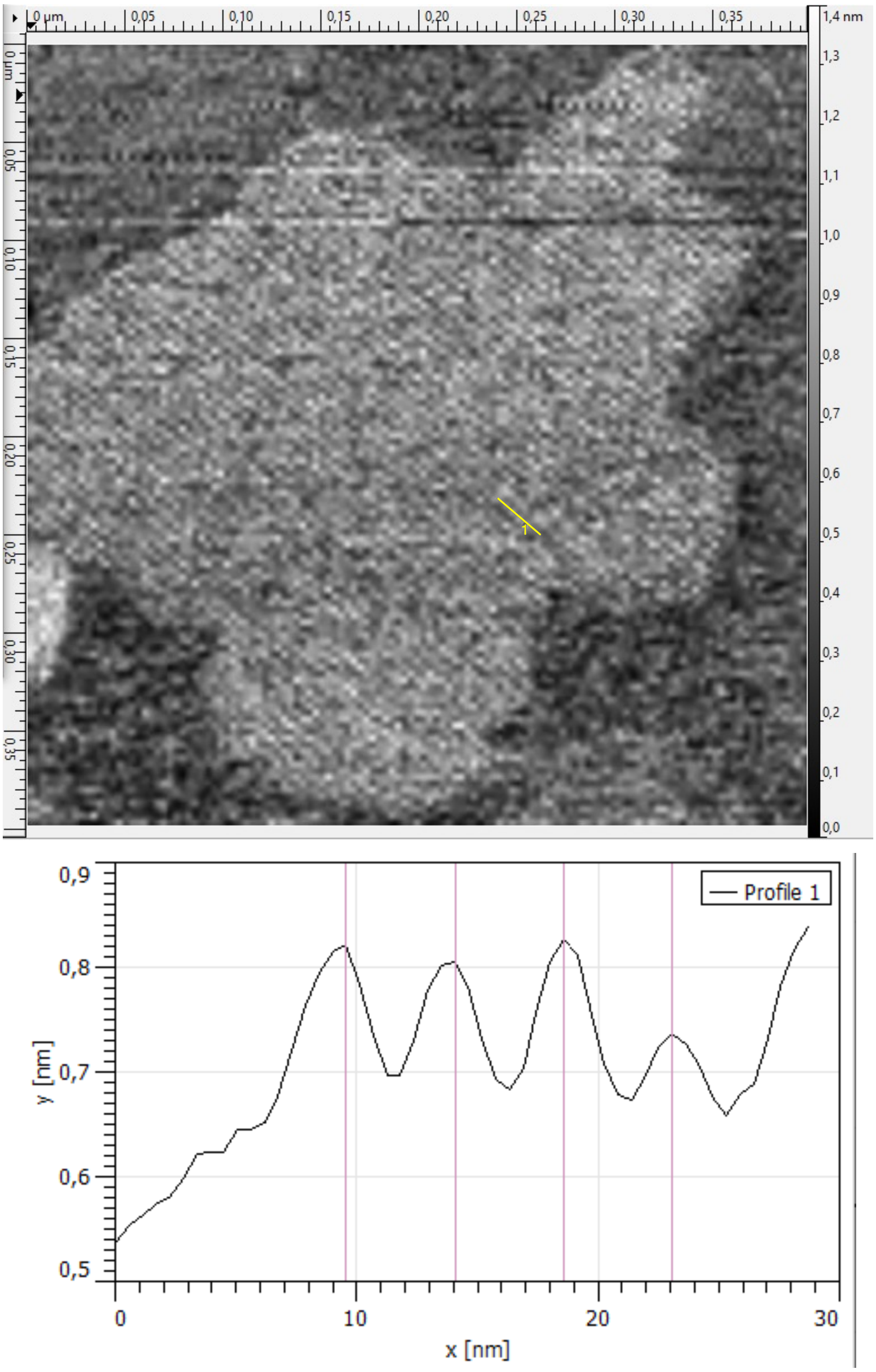

Points	X [nm]	Y [pm]	Length [nm]	Height [pm]	Angle [deg]
	9,56	823,4			
	14,07	805,7	4,51	-17,7	-0,22
	18,57	827,5	4,50	21,8	0,28
	23,05	737,0	4,48	-90,5	-1,16

Figure S14. High resolution AFM images and corresponding height profiles (yellow line) showing the helical pitch in the 2D-crystal generated by spin-coating an *m*-poly-(*R*)-1 $CHCl_3$ solution and using HOPG as substrate.

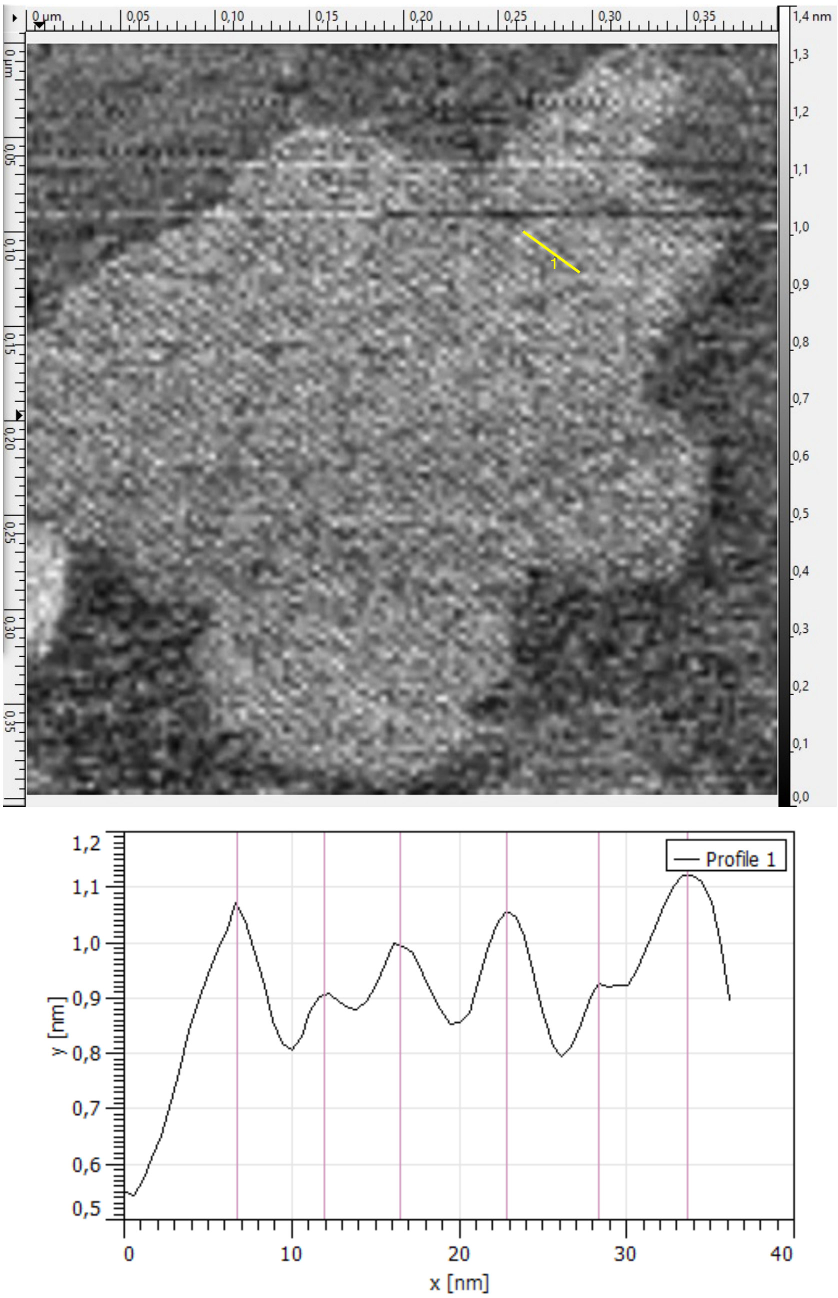

Points	X [nm]	Y [pm]	Length [nm]	Height [pm]	Angle [deg]
	6,71	1072,5			
	11,97	907,6	5,26	-165,0	-1,80
	16,47	997,5	4,51	89,9	1,14
	22,81	1061,2	6,34	63,7	0,58
	28,38	929,5	5,56	-131,8	-1,36
	33,62	1124,9	5,24	195,4	2,14

Figure S15. High resolution AFM images and corresponding height profiles (yellow line) showing the helical pitch in the 2D-crystal generated by spin-coating an *m*-poly-(*R*)-1 $CHCl_3$ solution and using HOPG as substrate.

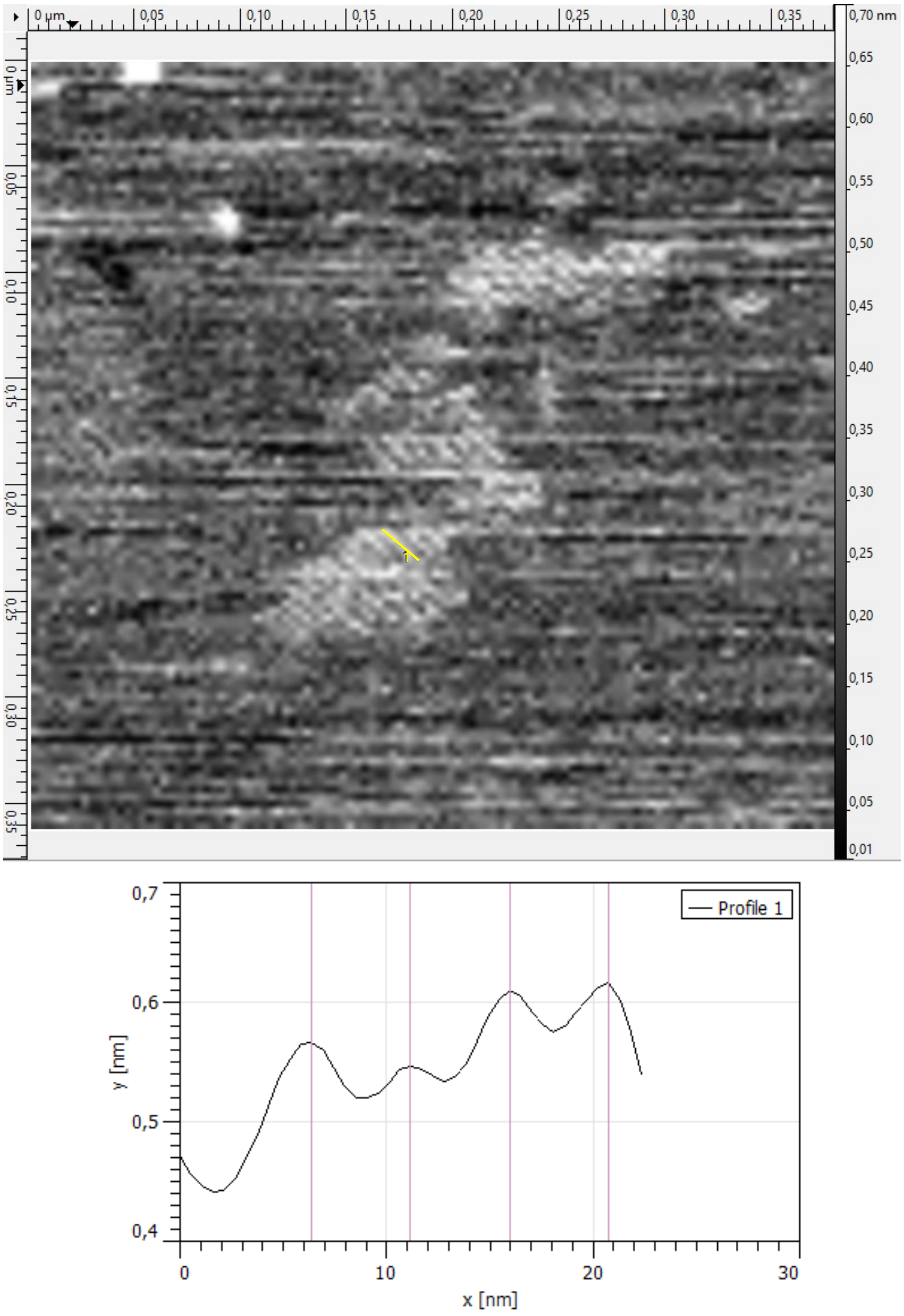

Points	X [nm]	Y [pm]	Length [nm]	Height [pm]	Angle [deg]
	6,38	568,0			
	11,17	547,6	4,79	-20,4	-0,24
	15,99	611,1	4,83	63,5	0,75
	20,75	617,4	4,76	6,3	0,08

Figure S16. High resolution AFM images and corresponding height profiles (yellow line) showing the helical pitch in the 2D-crystal generated by spin-coating an *m*-poly-(*R*)-1 $CHCl_3$ solution and using HOPG as substrate.

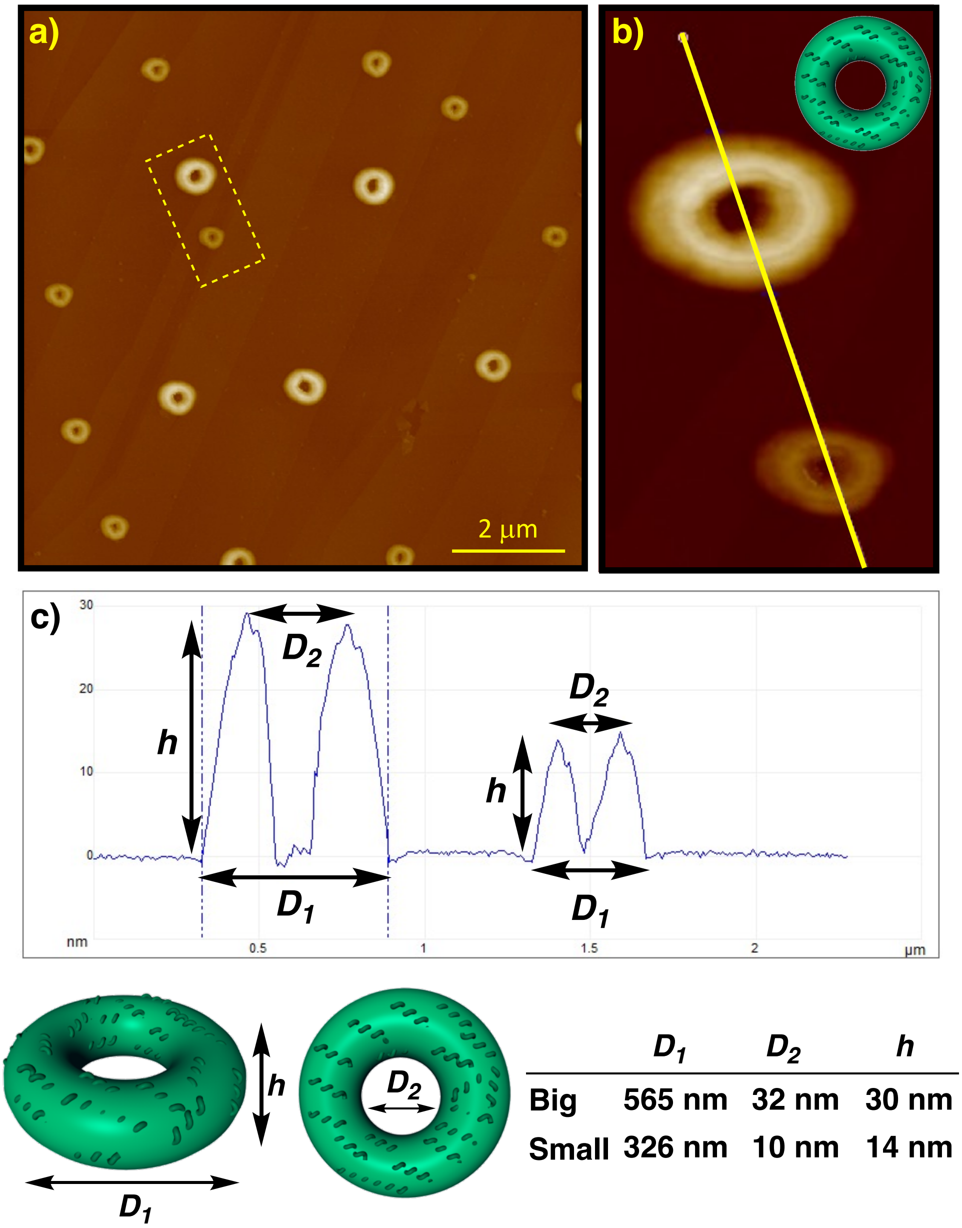

	D_1	D_2	h
**Big**	**565 nm**	**32 nm**	**30 nm**
**Small**	**326 nm**	**10 nm**	**14 nm**

Figure S17. (a) High resolution AFM images showing toroidal nanostructures self-assembled from of *m*-poly-(*L*)-**1** (HOPG as substrate). (b) Magnification of the area highlighted in picture (a) and (c) height profiles (yellow line) in picture (a). $CHCl_3$ solution spin-coated onto HOPG.

Computational Details

Considering the difficulties to carry out theoretical calculations on large polymers, we resorted to the use of a reduced-size representative oligomer with 20 monomer repeating units. The input structure used for the ECD calculations was adjusted according to the results obtained from structural techniques such as Raman, IR and AFM. A 3D structure 20-mer oligomer, with ω_1, ω_2 and ω_3 dihedral angles equal to -165°, 180° and 40° respectively, was obtained and submitted for ECD calculations (see Figure 4d in manuscript for dihedral angle definitions). The choice of the methodology used to carry out the ECD and UV calculations was determined by the size of the polymers under investigation. Considering this, time-dependent density functional theory (TD-DFT, Ref. S4) was the only option available, and we used it together with the CAM-B3LYP density functional (Ref. S5) and the 3-21G basis set (Ref. S6). We included 80 excitation energies in the calculation. This combination of density functional and basis set was selected based on density functional and basis set selection studies carried out in previous work, where it provided ECD and UV spectra for polyphenylacetylenes (PPAs) in an efficient way in terms of computational cost and reproducibility of the experimental spectra (Ref. S6 and S7). The use of larger bases results prohibitive in the case of the considered polymers. For a more efficient correlation between the theoretical and the experimental results and considering the known differences of DFT in getting accurate ECD spectra, we adjusted the theoretical 20-mer spectrum with a correlation factor obtained from comparison (between theory and experiment) of the wavelength and intensity at the maximum corresponding to the first Cotton effect. In this way, we evaluated a correction factor for lambda as the difference between the theoretical and experimental wavelengths, and we shifted the rest of the theoretical spectrum accordingly. Regarding the intensity, from the above comparison we rescaled the theoretical values to get the experimental intensity at the first Cotton effect band. To plot the ECD spectrum we selected a full width at half height (FWHM) of 43 nm and employed lorentzian curves.

Supporting References

S1. Leiras, S.; Freire, F.; Seco, J. M.; Quiñoá, E.; Riguera, R. Controlled modulation of the helical sense and the elongation of poly(phenylacetylene)s by polar and donor effects. *Chem. Sci.* **2013**,*4,* 2735-2743.

S2. Tarrío, J. J.; Rodríguez, R.; Fernández, B.; Quiñoá, E.; Freire, F. Dissymmetric Chiral Poly (diphenylacetylene) s: Secondary Structure Elucidation and Dynamic Luminescence. *Angew. Chem. Int. Ed.*, **2022**, *61*, e202115070.

S3. Rey-Tarrío, F.; Rodríguez, R.; Quiñoá, E.; Riguera, R.; Freire, F. Photochemical Electrocyclization of Poly(phenylacetylene)s: Unwinding Helices to Elucidate their 3D Structure in Solution. *Angew. Chem. Int. Ed.* **2021**, *60*, 8095-8103.

S4. Runge, E.; Gross, E. K. U. Density-Functional Theory for Time-Dependent Systems. *Phys. Rev. Lett.* **1984**, *52*, 997–1000.

S5. Yanai, T.; Tew, D. P.; Handy, N. C. A New Hybrid Exchange-Correlation Functional Using the Coulomb-Attenuating Method (CAM-B3LYP). *Chem. Phys. Lett.* **2004**, *393*, 51–57.

S6. Binkley, J. S.; Pople, J. A.; Hehre, W. J. Self-consistent molecular orbital methods. 21. Small split-valence basis sets for first-row elements. *J. Am. Chem. Soc.* **1980,** *102*, 939-947

S7. Fernández, B.; Rodríguez, Quiñoá, E.; Riguera, R.; Freire, F. Predicting the Helical Sense of Poly(phenylacetylene)s from their Electron Circular Dichroism Spectra. *Angew. Chem. Int. Ed*. **2018**, 57, 3666-3670.

S8. Fernández, B.; Rodríguez, Quiñoá, E.; Riguera, R.; Freire, F. Decoding the ECD Spectra of Poly(phenylacetylene)s: Structural Significance. *ACS Omega*, **2019**, 4, 3, 5233–5240.
